# Isomerisation of *nido*-[C_2_B_10_H_12_]^2–^ dianions: unprecedented rearrangements and new structural motifs in carborane cluster chemistry†
†Electronic supplementary information (ESI) available: Movies of the **1,7** to **7,9** isomerisation (17-79_isom.avi), *ortho*-carborane reduction (*ortho*-carborane_RED.avi) and *meta*-carborane reduction (*meta*-carborane_RED.avi); alternative reaction profiles; computed Cartesian coordinates and energies for all structures. See DOI: 10.1039/c5sc00726g



**DOI:** 10.1039/c5sc00726g

**Published:** 2015-03-24

**Authors:** David McKay, Stuart A. Macgregor, Alan J. Welch

**Affiliations:** a Institute of Chemical Sciences , School of Engineering and Physical Sciences , Heriot-Watt University , Edinburgh , EH14 4AS , UK . Email: dm228@st-andrews.ac.uk ; Fax: +44 (0)1314513180 ; Tel: +44 (0)1314518031

## Abstract

Dianionic *nido*-[C_2_B_10_]^2–^ species are key intermediates in the polyhedral expansion from 12- to 13-vertex carboranes and metallacarboranes, and the isomer adopted by these *nido* intermediates dictates the isomeric form of the 13-vertex product. Upon reduction and metallation of *para*-carborane up to five MC_2_B_10_ metallacarboranes can be produced (*Angew. Chem., Int. Ed.*, 2007, **46**, 6706), the structures of which imply the intermediacy of **1,7**-, **3,7**-, **4,7**-, **7,9**- and **7,10**-isomers of the *nido*-[C_2_B_10_]^2–^ species. In this paper we use density functional theory (DFT) calculations to characterise the reduction of *closo*-C_2_B_10_H_12_ carboranes and the subsequent isomerisations of the *nido*-[C_2_B_10_H_12_]^2–^ dianions. Upon reduction *para*-carborane initially opens to [**1,7**-*nido*-C_2_B_10_H_12_]^2–^ (abbreviated to **1,7**) and [**4,7**-*nido*-C_2_B_10_H_12_]^2–^ (**4,7**) and isomerisation pathways connecting **1,7** to **7,9**, **4,7** to **7,10** and **1,7** to **3,7** have been characterised. For *ortho*- and *meta*-carborane the experimental reduction produces **7,9** in both cases and computed pathways for both processes are also defined; with *ortho*-carborane rearrangement occurs *via* **7,8**, whereas with *meta*-carborane **7,9** is formed directly. The **7,9** isomer is the global minimum *nido*-structure. The characterisation of these isomerisation processes uncovers intermediates that adopt new structural motifs that we term *basket* and *inverted nido*. *Basket* intermediates feature a two-vertex basket handle bridging the remaining 10 vertices; *inverted nido* intermediates are related to known *nido* species, in that they have 5- and 6-membered belts, but where the latter, rather than the former, is capped, leaving a 5-membered open face. These new intermediates exhibit similar stability to the *nido* species, which is attributed to their relation to the 13-vertex docosahedron through the removal of 5-connected vertices. Isomerisation pathways starting from *nido* geometries are most often initiated by destabilisation of the cluster through a DSD process causing the 3-connected C^7^ vertex to move into a 4-connected site and a neighbouring B vertex to become 3-connected. The ensuing rearrangement of the cluster involves processes such as the pivoting of a 4-vertex diamond about its long diagonal, the pivoting of two 3-vertex triangles about a shared vertex and DSD processes. These processes are all ultimately driven by the preference for carbon to occupy low-connected vertices on the open 6-membered face of the resulting *nido* species.

## Abstract 2

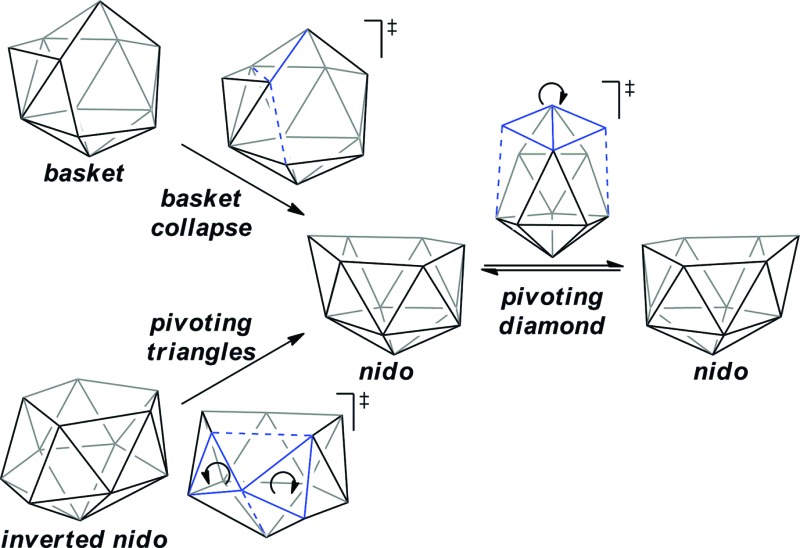
The formation and isomerisation of *nido*-[C_2_B_10_H_12_]^2–^ species is investigated through DFT calculations, which reveal novel *basket* and *inverted nido* intermediates and unusual inverconversion pathways, including *basket collapse* and *pivoting triangles* and *diamonds*.

## Introduction

**Fig. 1:**
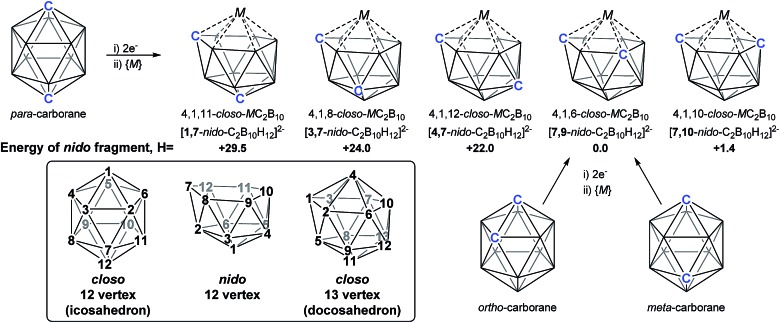
Results of polyhedral expansion of 12-vertex carboranes to 13-vertex metallacarboranes. For *para*-carborane {M} = {Ru(*p*-cymene)}, {C} = {CPh} relating to ref. 4; for *ortho*-carborane, *e.g.* {M} = {Ru(*p*-cymene)} or {CoCp}, {C} = *e.g.* {CH}.^2,5^ Computed energies of [*nido*-C_2_B_10_H_12_]^2–^ species given relative to **7,9** in kcal mol^–1^. See inset for formal numbering.

As predicted by Wade's Rules,^1^ the addition of a *skeletal electron pair* (SEP) to a *closo* polyhedron (with [*n* + 1] SEPs, where *n* is the number of vertices) results in the formation of the corresponding *nido* cluster ([*n* + 2] SEPs). A key synthetic route that relies upon this is the polyhedral expansion method, whereby 2-electron reduction of a *closo* precursor (normally a 12-vertex carborane) results in the formation of a *nido* fragment, which can then be capitated with a {BR} or {M} fragment. Application of this method led to both the first 13-vertex metallacarborane^2^ and the first 13-vertex carborane.^3^ In 2007 we showed that the polyhedral expansion of a single carborane precursor, [1,12-Ph_2_-1,12-*closo*-C_2_B_10_H_10_], with {M} = {Ru(*p*-cymene)} led to the formation of five isomeric supraicosahedral metallacarboranes of the form RuC_2_B_10_ (Fig. 1).^4^ This implies the presence of five isomeric *nido* fragments following reduction, *i.e.* **1,7**-, **3,7**-, **4,7**-, **7,9**- and [**7,10**-Ph_2_-**7,10**-*nido*-C_2_B_10_H_10_]^2–^. A concurrent computational study on [1,12-*closo*-C_2_B_10_H_12_] (*para*-carborane) suggested the first *nido*-fragments formed upon reduction were [**1,7**-*nido*-C_2_B_10_H_12_]^2–^ (termed **1,7** in the following) and [**4,7**-*nido*-C_2_B_10_H_12_]^2–^ (**4,7**). At the time, the further isomerisation of these species to the remaining *nido* species was not considered, however computing these directly provided relative energies, *H* (enthalpies, 0 K) of +29.5, +24.0, +22.0 and +1.4 kcal mol^–1^ for **1,7**, **3,7**, **4,7** and **7,10** respectively, all relative to **7,9** at 0.0 kcal mol^–1^. In contrast, related experimental studies adopting *ortho*- or *meta*-carborane precursors show that **7,9** is the only *nido* species formed.^2,5–7^

**Scheme 1:**
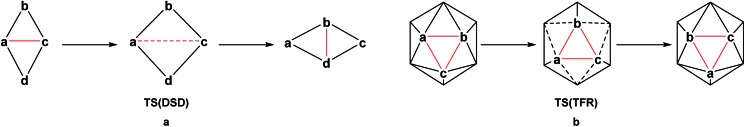
Neutral (hetero)borane isomerisation processes (a) DSD and (b) TFR.

The thermal rearrangement of 12-vertex *closo* (hetero)boranes has been the subject of continued investigation. Starting from *ortho*-carborane, the conversion to *meta*-carborane at 450 °C and then to *para*-carborane at 700 °C has been known for over 50 years and indeed was the route to their first syntheses.^8–10^ The processes involved in their rearrangement have been studied for many years, both theoretically^11–16^ and experimentally through labelling studies.^17–19^ In 1966, Lipscomb^13^ introduced the *diamond-square-diamond* (DSD) mechanism (Scheme 1a), while in the same year, Zakharkin and Kalinin^16^ suggested the *triangular face rotation* (TFR) mechanism, which can also be described as three concerted DSD processes (Scheme 1b). The DSD process in particular has since been recognised as key to carborane rearrangement. Wales^15^ adopted an eigenvector following method to map the potential energy surface of C_2_B_10_H_12_ at the Hartree–Fock (HF) level. Multi-step DSD-derived processes were found to dominate, however two TFR-type pathways were also located. High symmetry processes involving multiple simultaneous DSDs (*e.g.* the hextuple DSD process leading to a cuboctahedral geometry suggested by Lipscomb^13^) were discounted due to their unfeasibly high activation energies. Later studies also demonstrate an energetic preference for low symmetry processes.^11,12,14^ Wales also ruled out the *closo-nido-closo* rearrangement pathway, which requires opening of the cage to a high energy *pseudo-nido* intermediate.

More recently, Brown and McKee^11^ showed, through density functional theory (DFT) calculations, that a single step TFR process was favoured in the *ortho*- to *meta*-carborane isomerisation, while a two-step DSD pathway was preferred for isomerisation from *meta*- to *para*-carborane. Brown and McKee had discounted a two-step pathway from *ortho*- to *meta*-carborane, due to a high initial barrier between *ortho*-carborane and the intermediate involved. We later showed that the equivalent intermediate was formed upon the oxidation of **7,9** and characterised a lower energy process to form *ortho*-carborane, in agreement with experiment.^12^ Most recently, Sugden and co-workers^14^ investigated both of these isomerisation pathways through *ab initio* molecular dynamics (DFT-MD) adopting the PBE functional.

In stark contrast, few computational studies have involved reduced 12-vertex carboranes, despite the isomeric form of the reduced species ultimately dictating the isomer of the supraicosahedral product. McKee *et al.* ^20^ computed **7,9** directly, through HF calculations, for comparison with plausible protonated [*nido*-C_2_B_10_H_13_]^–^ structures. Later, Hermansson and co-workers^21^ showed in a study of the electron affinities of carboranes (also at the HF level) that sequential addition of two electrons to *meta*-carborane resulted in **7,9**, while geometries produced from reduction of *ortho*- and *para*-carborane showed only minor distortions and did not resemble *nido* fragments. More recently, 12-vertex *nido* carboranes and (bis)carboranes have featured in our investigations of the aforementioned oxidation of **7,9** to *ortho*-carborane,^12^ the room-temperature C–C activation of an arene at a 13-vertex metallacarborane,^22^ co-production of isomeric 13-vertex cobaltacarboranes from polyhedral expansion of a tethered carborane precursor^23^ and in the rational design of derivatives of **7,9** stabilised towards aerial oxidation.^24^

Herein we report a computational study of the isomerisation processes that follow from the initial 2e reduction of *para*-carborane and formation of **1,7** and **4,7**, revealing pathways interconnecting all five *nido* fragments inferred experimentally (see Fig. 1). The reduction of *ortho*-carborane is shown to initially produce **7,8**, before rearranging to the experimental product, **7,9**. *meta*-Carborane reduction proceeds to **7,9** directly, where the barrierless rearrangement process is rationalised by relation to the *nido* isomerisation pathways. In the completion of this work, we uncover and rationalise new dianionic 12-vertex carborane structures which we refer to as *basket* and *inverted nido* intermediates and characterise new, unexpectedly complex processes interconnecting *nido* species, ultimately linking all intermediates to the global minimum, **7,9**.

## Results

### 1. Formation of **1,7** and **4,7**

**Fig. 2:**
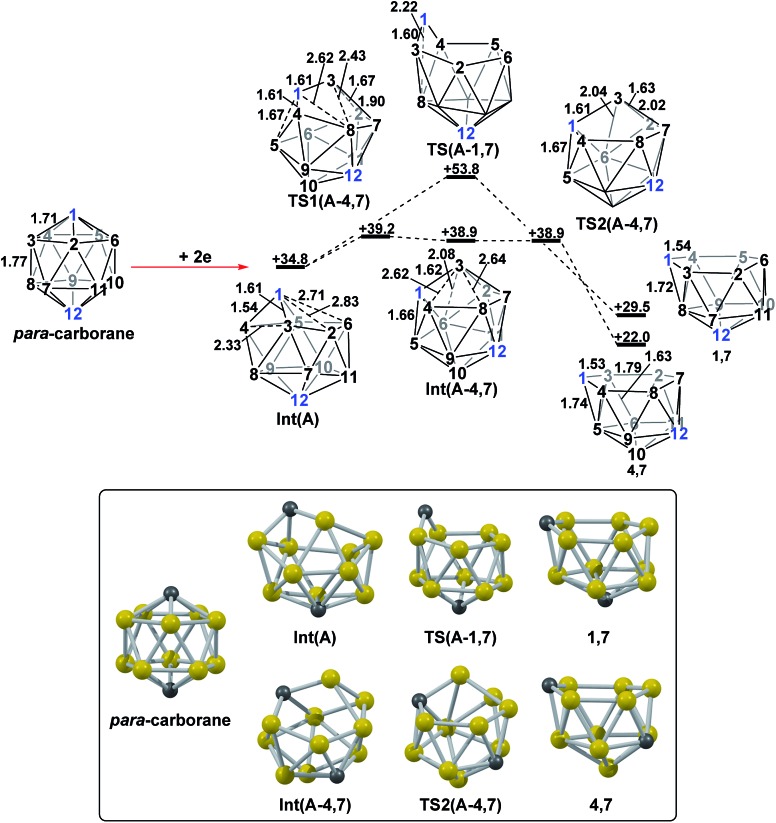
2e addition to *para*-carborane followed by isomerisation to the first *nido* intermediates, **1,7** and **4,7**. Numbering of CH vertices (blue) and BH vertices (black) consistent with *para*-carborane. See inset for key computed structures along the pathways. Selected distances in Å and energies relative to **7,9** in kcal mol^–1^. H atoms omitted for clarity.

**Fig. 3:**
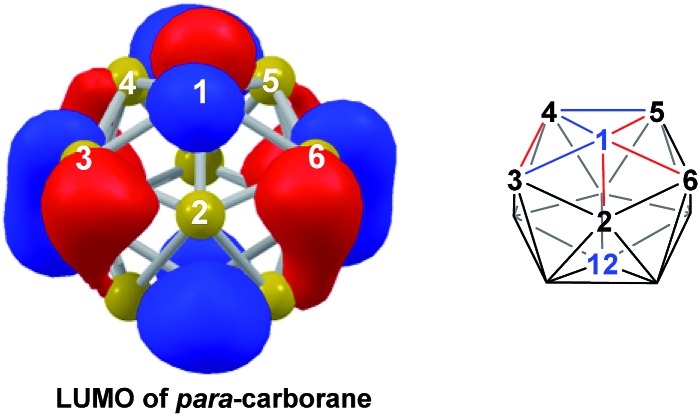
Left: representation of the LUMO of *para*-carborane at the BP86/6-31G** level; contour value = 0.05. Right: qualitative schematic shows bonding in blue and antibonding interactions in red. H atoms omitted for clarity.

DFT calculations were performed at the BP86/6-31G** level using Gaussian 03 and we report zero-point corrected electronic energies, *H*, for all computed species relative to **7,9** (see Computational details). In order to model the reduction of *para*-carborane, first the neutral geometry was optimised, then two electrons were added and the system was re-optimised. This resulted in an initial reduced minimum, **Int(A)** (*H* = +34.8 kcal mol^–1^; Fig. 2). **Int(A)** features 4-membered (B^3^–B^8^–B^4^–C^1^) and 5-membered (B^3^–C^1^–B^5^–B^6^–B^2^) open faces and is related to the *nido* geometry, **1,7**, by a DSD process in the C^1^–B^3^–B^8^–B^4^ face. This was characterised by decreasing the C^1^···B^8^ distance, giving **TS(A-1,7)** (*H* = +53.8 kcal mol^–1^). Through this process, C^1^, which is 3-connected in **Int(A)**, becomes 2-connected in the transition state and returns to 3-connected in **1,7**, giving a barrier of 19.0 kcal mol^–1^. Alternatively, movement of B^3^ across the 5-membered face towards B^6^ in **Int(A)** gives **Int(A-4,7)**, through a lower barrier of 4.4 kcal mol^–1^. **Int(A-4,7)** is topologically equivalent to **Int(A)** and features 4-membered C^1^–B^3^–B^2^–B^6^ and 5-membered C^1^–B^3^–B^7^–B^8^–B^4^ faces. We refer to such a topology as a *basket* intermediate (discussed in detail later) where here C^1^ and B^3^ vertices bridge the remaining 10 vertices in a way that resembles a basket handle. From **Int(A-4,7)** decreasing the B^3^···B^6^ distance causes the 4-membered face to close *via* **TS2(A-4,7)** (*H* = +38.9 kcal mol^–1^) in what is effectively a barrierless process. Thereafter, a DSD process occurs in the C^1^–B^3^–B^6^–B^5^ diamond, breaking the C^1^–B^6^ connection and forming the B^3^–B^5^ connection, allowing C^1^ to become 3-connected and furnishing the *nido* geometry, **4,7**. These energy profiles suggest that processes decreasing the number of connections to carbon vertices and increasing the number of connections to boron vertices are favoured. The lower barrier to formation of **4,7** than of **1,7** also suggests that processes involving movement of boron are easier than those involving movement of carbon. This is supported by electronegativity arguments; the radial orbitals of the carbon vertices, being more contracted than those of boron, do not allow stabilisation of higher-connected sites or longer connections. The movement of vertices from the initial *para*-carborane geometry to give **Int(A)** can be rationalised by visualisation of the LUMO of *para*-carborane (Fig. 3). This features a π-antibonding interaction along the C^1^–B^6^ connection and a further antibonding interaction between B^3^ and B^4^. Therefore the 2e occupation of this orbital is consistent with the breaking of these interactions to give a 4-membered C^1^–B^3^–B^8^–B^4^ and a 5-membered C^1^–B^3^–B^2^–B^6^–B^5^ face in **Int(A)**.

### 2. Onward isomerisations of **1,7** and **4,7**: general strategies

**Scheme 2:**
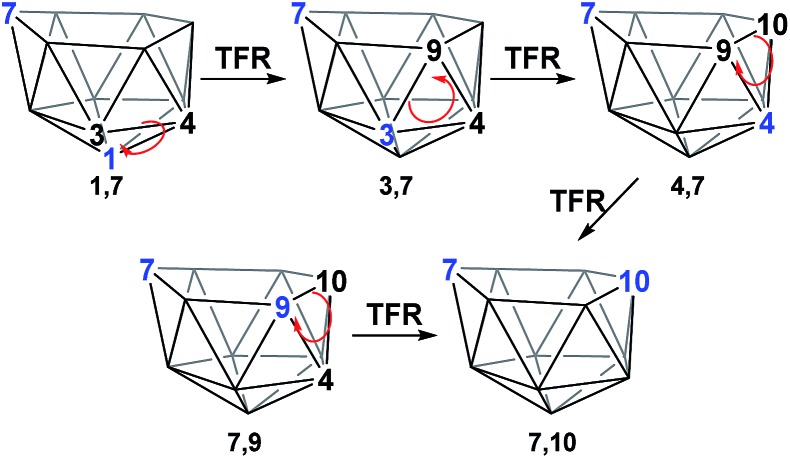
TFR processes considered to interconvert *nido* geometries.

The geometries of **1,7** and **4,7** were considered as starting points towards formation of the remaining *nido* species, **3,7**, **7,9** and **7,10**. Initially, we considered the possibility of TFR processes linking *nido* isomers (Scheme 2); in **1,7**, rotation of the C^1^–B^3^–B^4^ triangle could be envisaged to interconvert **1,7** and **3,7** and rotation of the same triangle again, or C^3^–B^9^–B^4^, would exchange **3,7** and **4,7**. Likewise, in **4,7**, rotation of the C^4^–B^9^–B^10^ triangle could give **7,9** and **7,10**. However, attempts to characterise such processes through potential energy surface searching (linear transits) were unsuccessful from either **1,7** or **4,7**. Several atoms had to be fixed in position in order to prevent non-targetted rearrangement of the cluster. This suggested that the TFR process, though relevant to *closo* carborane isomerisation, was higher in energy than other processes available to the more flexible *nido* species. It was noted that the lowest energy vibrational mode of all *nido* species computed involves rotation of the 6-membered face above the 5-membered 2–3–4–5–6 belt, where the largest displacement is seen in the 7-position. Taking the lead from the mode-following approach of Wales,^15^ we used the transition state (TS) optimisation option in Gaussian 03,^25^ which follows the lowest energy vibrational mode to a saddle-point on the PES, thus allowing low energy transition states to be sought *a priori*, direct from selected minima. The present C_2_B_10_H_12_ clusters (which lack polyatomic exopolyhedral substituents) lend themselves to mode-following since the lowest energy vibrational mode *always* involves displacement of cluster vertices and so is productive towards cluster rearrangement. Mode-following was therefore always attempted in the first instance for any transition state search (see Computational Details). From **1,7** and **4,7** this revealed surprising and contrasting isomerisation processes, which see rearrangement of **1,7** to **7,9** in a single step and **4,7** to **7,10** in a multi-step process.

#### 2.1 Formation of **7,9** *via* isomerisation of **1,7**

**Fig. 4:**
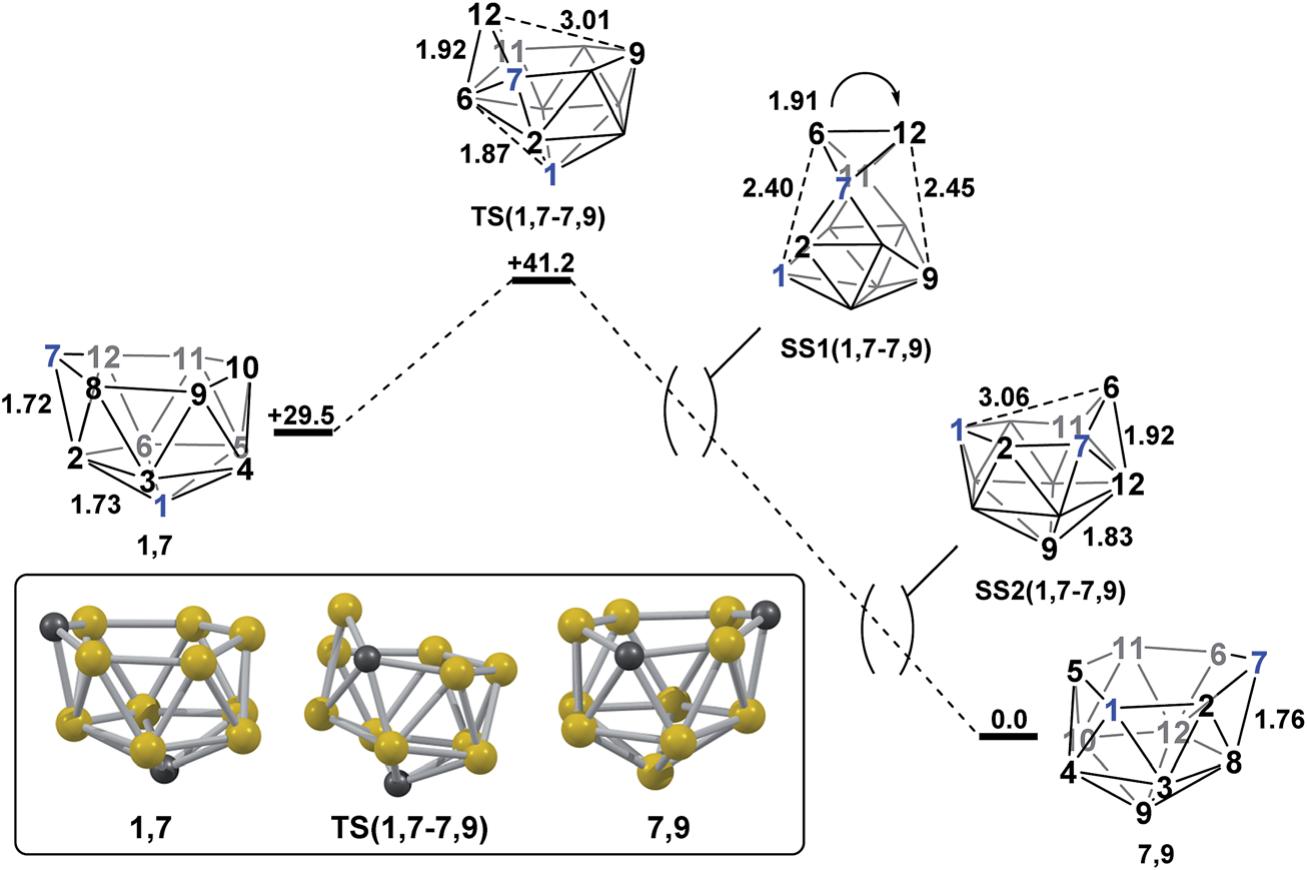
Isomerisation from **1,7** to **7,9**. Numbering of CH vertices (blue) and BH vertices (black) consistent with **1,7**. Inset shows the computed structure of **TS(1,7-7,9)**. Selected distances in Å and energies relative to **7,9** in kcal mol^–1^. H atoms omitted for clarity.

Mode-following from **1,7** provided a transition state, **TS(1,7-7,9)** (*H* = +41.2 kcal mol^–1^; Fig. 4), connecting **1,7** directly to **7,9**, through a barrier of just 11.7 kcal mol^–1^. **TS(1,7-7,9)** is formed through a DSD process in the C^7^–B^12^–B^6^–B^2^ diamond and exhibits a 3-connected boron vertex, B^12^, which protrudes from the 6-membered open face by 0.81 Å w.r.t. the C^7^–B^8^–B^9^–B^10^–B^11^ least-squares mean plane (for comparison, C^7^ protrudes by 0.28 Å and 0.27 Å from the open faces of **1,7** and **7,9** respectively). Visualisation of the single imaginary vibrational mode of **TS(1,7-7,9)** sees movement of B^12^ relative to the open face, where the B^12^···B^9^ distance is 3.01 Å at the transition state geometry. Characterisation of **TS(1,7-7,9)** *via* IRC calculations revealed a remarkable and unanticipated process in which the cluster inverts in one step from **1,7**, which has a CB_5_ 6-membered open face, to **7,9**, with a C_2_B_4_ open face. Fig. 4 shows the isomerisation process, with atom labelling consistent with the formal numbering of **1,7** to allow vertex movement to be followed; two snap-shots, **SS1(1,7-7,9)** and **SS2(1,7-7,9)**, are shown to further aid in visualising the process and a movie is provided in the ESI.† The initial movement away from **TS(1,7-7,9)** involves pivoting about the long C^7^···B^11^ diagonal of the C^7^–B^6^–B^11^–B^12^ diamond. **SS1(1,7-7,9)** illustrates the midpoint of this process. The pivoting continues, opening the C^1^–B^2^–C^7^–B^6^–B^11^–B^5^ face and closing the C^7^–B^8^–B^9^–B^10^–B^11^–B^12^ face of the starting structure. At **SS2(1,7-7,9)** the original open face has closed to give a geometry resembling a mirror image of **TS(1,7-7,9)**, but with both C-vertices now on the open face. Finally, a barrierless DSD process in the C^7^–B^6^–B^12^–B^8^ diamond moves C^7^ into the 3-connected site to give **7,9**.

#### 2.2 Formation of **7,10** *via* isomerisation of **4,7**

**Fig. 5:**
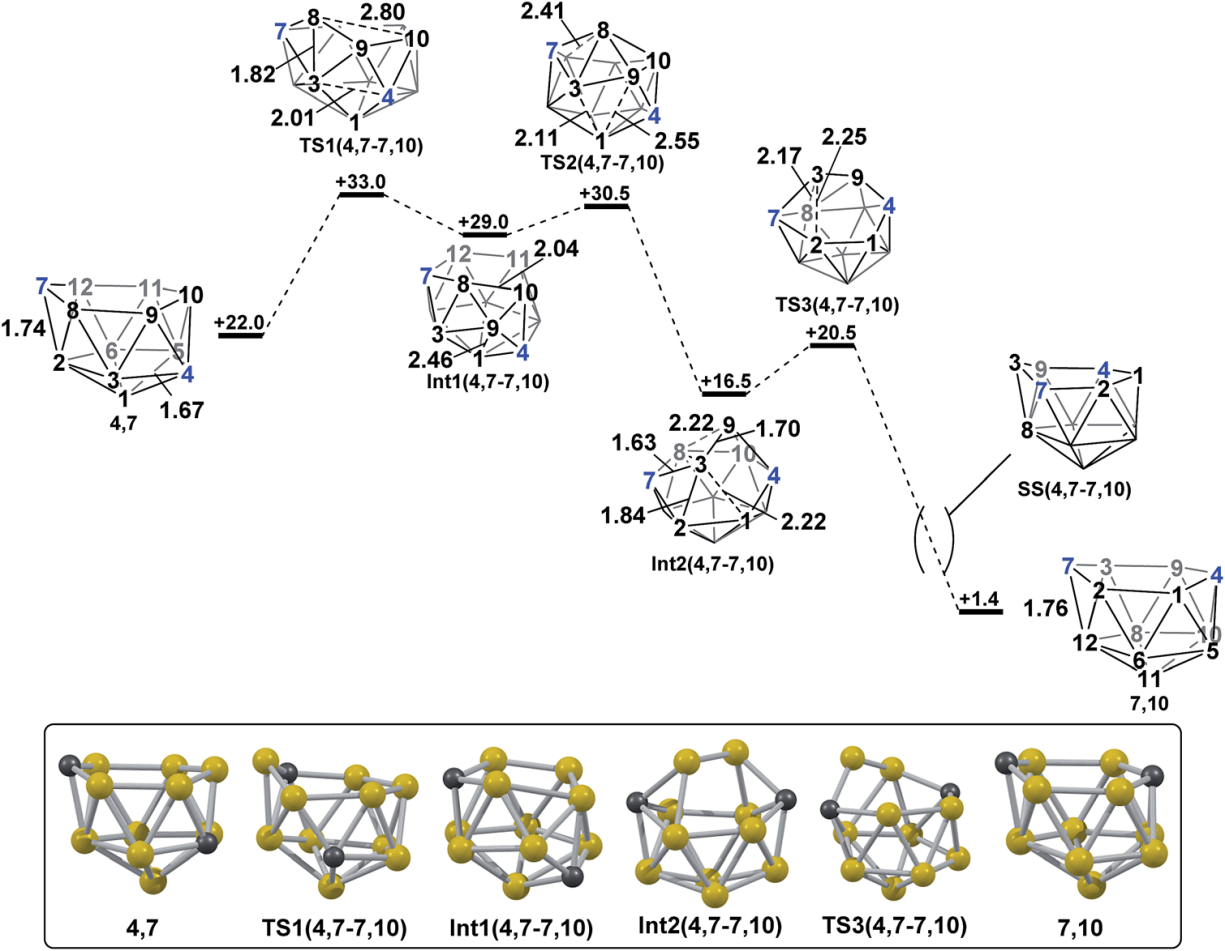
Isomerisation from **4,7** to **7,10**. Numbering of CH vertices (blue) and BH vertices (black) consistent with **4,7**. Inset shows key computed structures along the pathway. Selected distances in Å and energies relative to **7,9** in kcal mol^–1^. H atoms omitted for clarity.

In contrast, the isomerisation of **4,7** to **7,10** was found to be a multistep process (Fig. 5) which was characterised through sequential mode-following steps. The first transition state, **TS1(4,7-7,10)** (*H* = +33.0 kcal mol^–1^) again gives a low isomerisation barrier of 11.0 kcal mol^–1^. **TS1(4,7-7,10)** is related to **4,7** by a single DSD process in the C^7^–B^8^–B^3^–B^2^ diamond, resulting in a geometry topologically equivalent to **TS(1,7-7,9)**, exhibiting a 3-connected B^8^ vertex protruding from the open face by 0.56 Å. Further distortion of the cluster has also occurred, where the B^3^–C^4^ connection has lengthened from 1.67 Å to 2.01 Å, producing an open B^1^–B^3^–B^9^–C^4^ diamond. The imaginary mode associated with **TS1(4,7-7,10)** displays rotation of the B^3^–B^8^–B^9^ and C^4^–B^9^–B^10^ triangles, giving a hinging motion about the shared B^9^ vertex. Towards **Int1(4,7-7,10)** (*H* = +29.0 kcal mol^–1^), this motion further elongates the B^3^–C^4^ connection to 2.57 Å, shortens the B^9^–B^1^ distance to 2.46 Å and sees formation of an incipient B^8^–B^10^ connection of 2.04 Å, giving a 5-membered C^7^–B^8^–B^10^–B^11^–B^12^ face. Mode-following from **Int1(4,7-7,10)** gives **TS2(4,7-7,10)** (*H* = +30.5 kcal mol^–1^) in which B^8^ has moved above the open face and the B^8^–B^3^ and B^8^–B^9^ connections are lengthened, but not broken, from 1.80 Å and 1.68 Å respectively in **Int1(4,7-7,10)** to 1.90 Å and 1.74 Å respectively in **TS2(4,7-7,10)**. Similarly, the long B^1^···B^3^ distance is lengthened further from 1.99 Å in **Int1(4,7-7,10)** to 2.11 Å in the transition state. From **TS2(4,7-7,10)** to **Int2(4,7-7,10)** the movement of B^8^ across the larger face is continued, leading to formation of connections B^8^–B^12^ and B^8^–B^11^ in **Int2(4,7-7,10)** while breaking the B^3^–B^8^ and B^9^–B^8^ connections. *C*_2_ symmetric **Int2(4,7-7,10)** (*H* = +16.5 kcal mol^–1^) is a further example of a *basket* intermediate with two equivalent 5-membered CB_4_ faces, where the B^3^–B^9^ edge comprises the basket handle and the C vertices adopt bridgehead positions, 4 and 7. It is also relatively thermodynamically stable, having a lower energy than the starting *nido* species, **4,7**. However, it is kinetically unstable due to the low barrier to its subsequent rearrangement. Rearrangement of **Int2(4,7-7,10)** to give a *nido* fragment is equivalent to that found for **Int(A)**. Mode-following gives **TS3(4,7-7,10)** (*H* = +20.5 kcal mol^–1^), in which the B^3^–B^2^ connection is lengthened from 1.84 Å to 2.25 Å at the transition state and the B^9^–B^8^ connection shortens to 2.02 Å, giving a structure that closely resembles **TS(A-1,7)** and **TS2(A-4,7)**. In visualising the IRC calculations from **TS3(4,7-7,10)** B^3^ is initially seen to remain 3-connected, however a DSD process in the B^3^–B^8^–B^12^–C^7^ diamond gives a 3-connected C^7^. Overall this transformation sees **4,7** rearrange to **7,10** where the former C^4^ vertex now adopts the C^10^ position and the B^3^–B^8^–B^9^ triangle is effectively transferred across the open face of the cluster in order for this to be accomplished. The process is exothermic with Δ*H* = –20.6 kcal mol^–1^.

#### 2.3 Formation of **3,7** *via* isomerisation of **1,7**

**Fig. 6:**
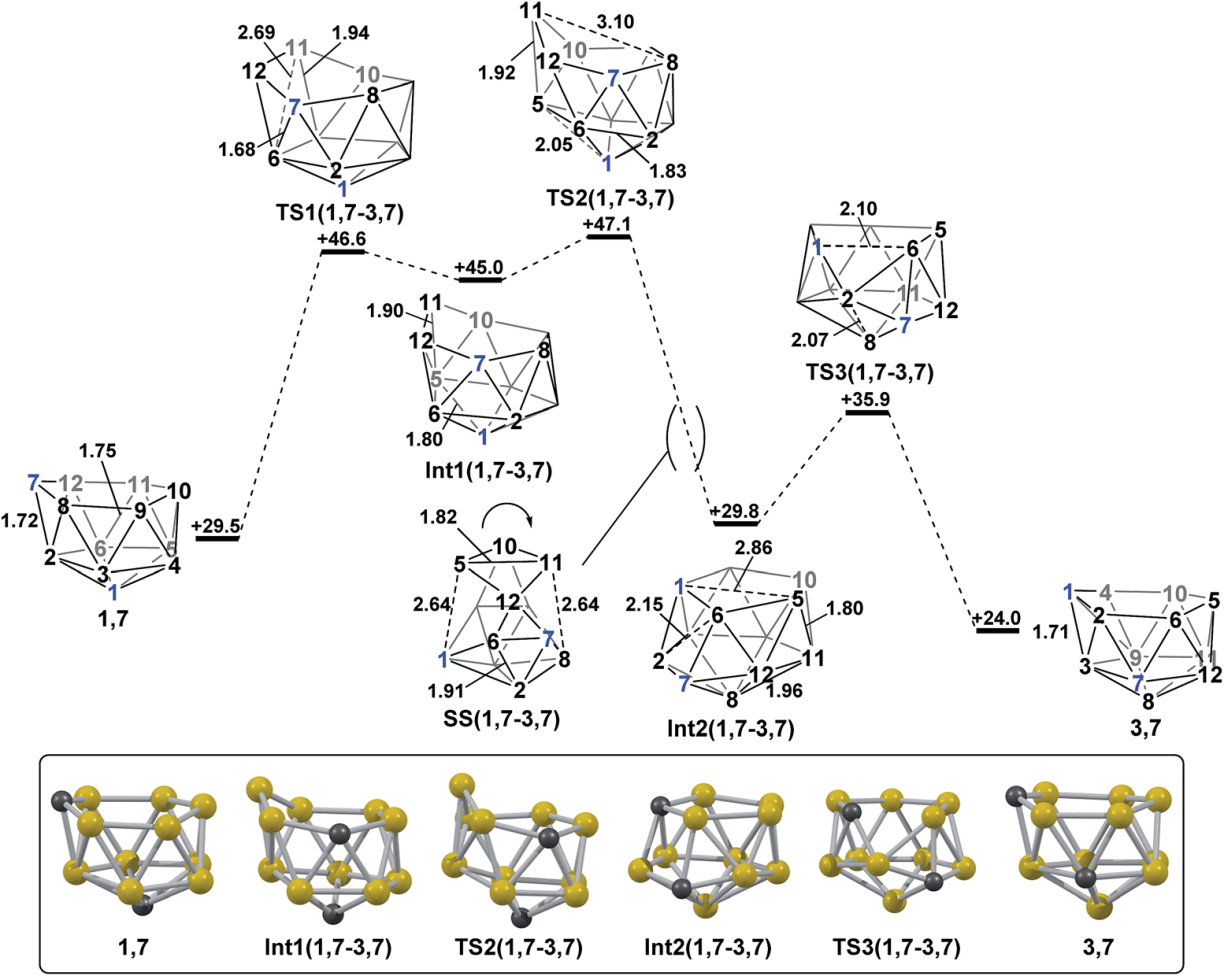
Isomerisation from **1,7** to **3,7**. Numbering of CH vertices (blue) and BH vertices (black) consistent with **1,7**. Inset shows key computed structures along the pathway. Selected distances in Å and energies relative to **7,9** in kcal mol^–1^. H atoms omitted for clarity.

The final remaining target *nido* fragment, **3,7**, lies between **1,7** and **4,7** in energy (*H* = +24.0 kcal mol^–1^, Fig. 6). It was therefore considered most likely to form in an exothermic process starting from **1,7**. As the mode-following approach had previously defined the pathway from **1,7** to **7,9** (and not **3,7**) we focussed on the reverse process, from **3,7** to **1,7**. In this case, however, the lowest mode led to a facile, degenerate, two-step DSD process that links equivalent forms of **3,7** (Δ*H* ^‡^ = 6.2 kcal mol^–1^; see ESI Fig. S1†). Instead a linear transit shortening the C^1^ to B^6^ distance allowed us to locate a new minimum, **Int2(1,7-3,7)**, that was comparable in energy to **1,7** and **3,7**. **Int2(1,7-3,7)** is topologically equivalent to **Int1(4,7-7,10)**. Therefore a process analogous to that linking **4,7** to **Int1(4,7-7,10)**, where two triangle pivot about a shared vertex, was sought through STQN calculations. This resulted in location of **TS3(1,7-3,7)**, through which the C^1^–B^3^–B^2^ and B^2^–B^6^–C^7^ triangles (as numbered in Fig. 6) pivot about B^2^. The remaining challenge was then to connect **Int2(1,7-3,7)** to **1,7**. **Int2(1,7-3,7)** features one 5-membered face including C^1^. By analogy now with **TS(1,7-7,9)**, it was envisaged that a process pivoting the B^12^–B^5^–B^10^–B^11^ diamond about its long B^12^···B^10^ diagonal would provide the **1,7** geometry. Such a process, located by decreasing the C^1^···B^5^ distance with the B^5^–B^11^ distance frozen, gave **TS2(1,7-3,7)** (*H* = +47.1 kcal mol^–1^). A snap-shot geometry, **SS(1,7-3,7)** is given in Fig. 6 to illustrate this process, where the B^5^–B^10^–B^11^–B^12^ diamond pivots about the long B^10^···B^12^ diagonal. This gives (in the reverse direction) **Int1(1,7-3,7)** (*H* = +45.0 kcal mol^–1^). Mode-following from **Int1(1,7-3,7)** gave **TS1(1,7-3,7)** directly, emphasising the ability of this technique to give the lowest energy transition state associated with a minimum, *i.e.* here locating the lower **TS1(1,7-3,7)** at Δ*H* = +1.6 kcal mol^–1^ rather than the slightly higher **TS2(1,7-3,7)** at Δ*H* = +2.1 kcal mol^–1^ w.r.t. **Int1(1,7-3,7)**. The process linking **1,7** to **Int1(1,7-3,7)** involves a double-DSD step, where the first DSD (in the C^7^–B^2^–B^6^–B^12^ diamond) has already occurred at **TS1(1,7-3,7)** and the second furnishes **Int1(1,7-3,7)**. The overall isomerisation process forming **3,7** from **1,7** has a barrier of 17.6 kcal mol^–1^, corresponding to **TS2(1,7-3,7)**, and is exothermic by Δ*H* = –5.5 kcal mol.

#### 2.4 Remaining isomerisation pathway; **7,10** to **7,9**

**Fig. 7:**
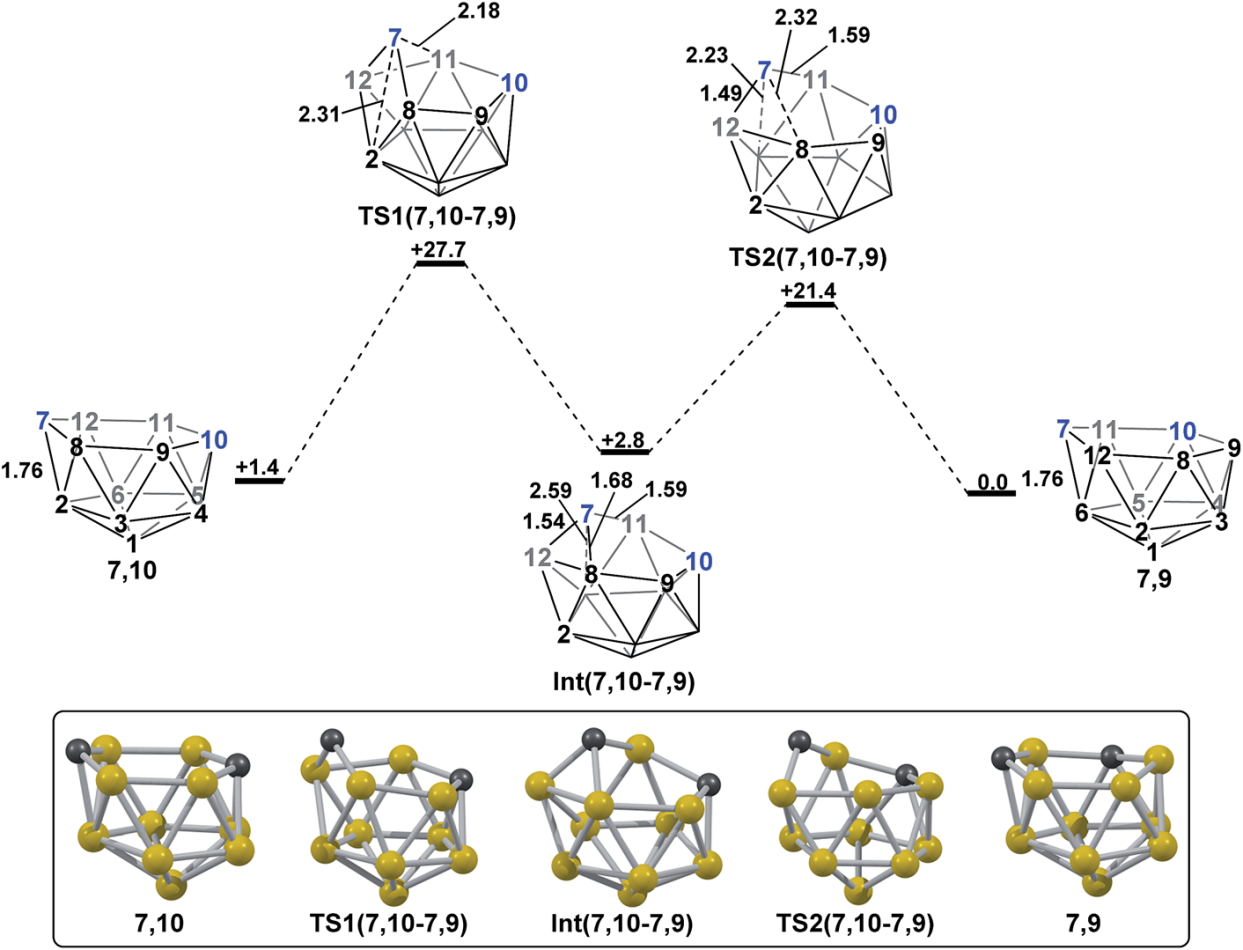
Isomerisation from **7,10** to **7,9**. Numbering of CH vertices (blue) and BH vertices (black) consistent with **7,9**. Inset shows key computed structures along the pathway. Selected distances in Å and energies relative to **7,9** in kcal mol^–1^. H atoms omitted for clarity.

At this stage isomerisation processes have been characterised that rationalise the formation of all five *nido* species targeted. However, for completeness, it is desirable to connect all species to **7,9**, the global minimum. **1,7** connects to **7,9** directly, whereas **3,7** connects to **7,9** *via* **1,7** and **4,7** connects to **7,9** *via* **7,10** (see Discussion section). The remaining isomerisation, from **7,10** to **7,9**, is discussed below and shown in Fig. 7. Starting from **7,10**, mode-following results in a degenerate process where the 3-connected C^7^ becomes 4-connected and B^8^ and B^9^ (or equivalent B^12^ and B^11^) become 3-connected. This is similar to the initial movement of vertices seen for the formation of **3,7** from **1,7**, however here it does not lead to an isomerisation process. A linear transit was therefore adopted, increasing the C^7^–B^2^ distance, to cause a DSD process in the C^7^–B^8^–B^2^–B^12^ diamond. This gave **TS1(7,10-7,9)** (*H* = +27.7 kcal mol^–1^) which leads to a *basket* intermediate, **Int(7,10-7,9)** (*H* = +2.8 kcal mol^–1^), where C^7^–B^11^ forms the basket handle. **Int(7,10-7,9)** is related to **7,9** through a DSD process in the C^7^–B^11^–B^6^–B^12^ diamond, which was characterised through location of **TS2(7,10-7,9)** (*H* = +21.4 kcal mol^–1^). The isomerisation from **7,10** to **7,9** therefore involves two DSD processes, through a *basket* intermediate, with an overall barrier of 26.3 kcal mol^–1^.

### 3. Reductions of *ortho*- and *meta*-carborane

**Fig. 8:**
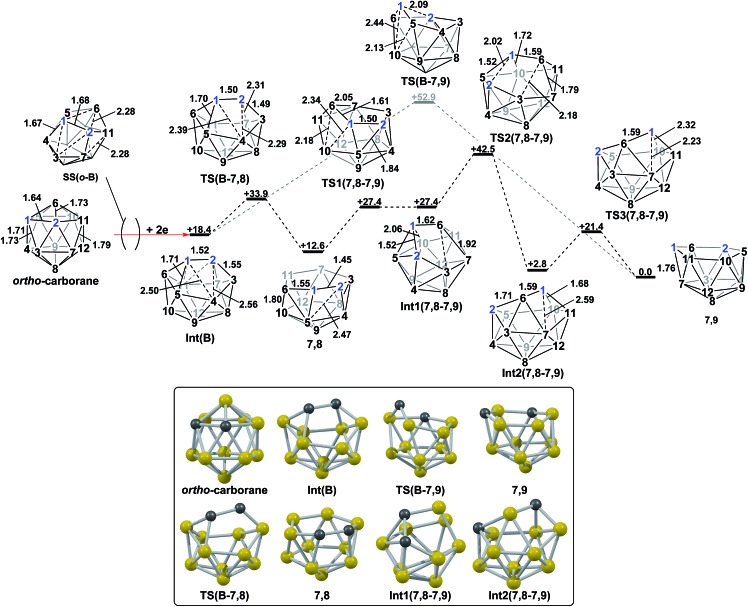
2e addition to *ortho*-carborane followed by isomerisation to **7,9** through a one-step pathway (grey) or a multi-step pathway, *via* **7,8** (black). Numbering of CH vertices (blue) and BH vertices (black) consistent with *ortho*-carborane. Inset shows key computed structures along the pathway. Selected distances in Å and energies relative to **7,9** in kcal mol^–1^. H atoms omitted for clarity.

**Fig. 9:**
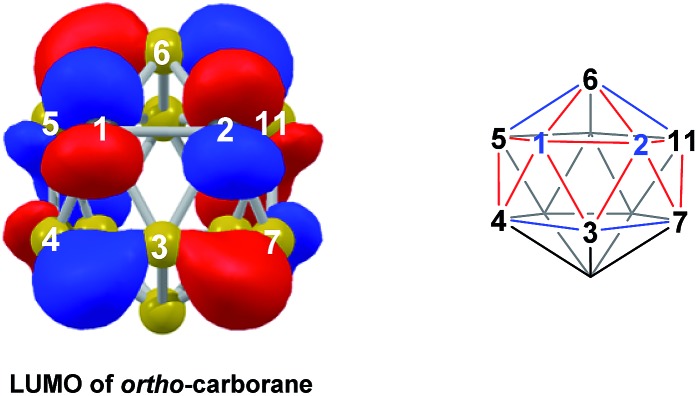
Left: LUMO of *ortho*-carborane at the BP86/6-31G** level; contour value = 0.05. Right: qualitative schematics show key bonding (blue) and antibonding (red) interactions. H atoms omitted for clarity.

The experimental reductions of *ortho*- and *meta*-carborane each lead to **7,9**.^2,5–7^ Hermansson *et al.* ^21^ showed in a study of carborane electron affinities that, at the HF level of theory, sequential addition of two electrons to *meta*-carborane produced a **7,9** *nido* geometry, whereas *ortho*-carborane was only slightly distorted. Through the present DFT calculations, we have now characterised the rearrangement processes undergone by both of these species following 2e reduction, ultimately giving **7,9**, which can be rationalised by relating them to the processes seen above. By analogy to the computational treatment of the reduction of *para*-carborane, 2e were added to the optimised geometry of *ortho*-carborane and the structure re-optimised as a dianion. This gave **Int(B)** (*H* = +18.4 kcal mol^–1^ above **7,9**; Fig. 8). **Int(B)** is another example of a *basket* intermediate, where here the geometry is *C*_2_ symmetric and the C^1^ and C^2^ vertices form the basket handle. The C^1^–C^2^ connection in **Int(B)** is shortened to 1.52 Å w.r.t. 1.64 Å in *ortho*-carborane, indicative of single bond character. From **Int(B)**, a *basket collapse* process was characterised through **TS(B-7,9)** (*H* = +52.9 kcal mol^–1^; see Fig. 8, grey pathway) and involves DSD processes in the C^1^–B^5^–B^10^–B^6^ diamond (with C^1^···B^10^ and B^5^···B^6^ distances of 2.44 Å and 2.58 Å respectively) and breaking of the C^1^–C^2^ connection to give **7,9** in a single step. However, this process exhibits a high overall barrier of 34.5 kcal mol^–1^, consistent with it being dominated by the breaking of a C–C connection with single bond character. An alternative *basket collapse* process was characterised through mode-following (Fig. 8, black pathway). This pathway initially maintains the C^1^–C^2^ connection, giving **7,8** through a low barrier of 15.5 kcal mol^–1^ in which the C^1^–C^2^ connection is shortened still further to 1.45 Å. Mode-following from **7,8** led to a degenerate rearrangement involving a DSD process in the C^1^–C^2^–B^4^–B^5^ diamond (as numbered in Fig. 8), forming a C^2^–B^5^ connection through a barrier of 14.8 kcal mol^–1^. Linear transits were therefore conducted to discover a pathway leading to **7,9**. A low energy transition state, **TS1(7,8-7,9)** (*H* = +27.4 kcal mol^–1^) was located, and provided a *basket* intermediate, **Int1(7,8-7,9)**. From here, C^1^–C^2^ bond breaking proceeds through **TS2(7,8-7,9)** at over 10 kcal mol^–1^ lower than **TS(B-7,9)** (*H* = +42.5 kcal mol^–1^), giving a barrier of 29.9 kcal mol^–1^ from **7,8**. This process leads to **Int2(7,8-7,9)** at *H* = +2.8 kcal mol^–1^, which is identical to **Int(7,10-7,9)**. Therefore the *basket collapse* process described above to give **7,9** is repeated in this pathway; here forming the C^1^–B^7^ connection through a DSD process in the C^1^–B^11^–B^7^–B^6^ diamond of the **Int2(7,8-7,9)** *basket*. The *nido*-**7,8** isomer is implicated experimentally in the synthesis if 4,1,2-MC_2_B_10_ species from *ortho*-carborane, where exopolyhedral hydrocarbyl or silyl tethers connecting the C-vertices ensure the C positions remain adjacent.^3,26,27^ With the removable silyl tether metallation with M = {CoCp} leads to the concurrent formation of the expected 4,1,2-MC_2_B_10_ species, but also the 4,1,6-MC_2_B_10_ isomer, indicating that isomerisation of the *nido*-**7,8** fragment to the **7,9** form is possible.^23^ The initial strengthening of the C^1^–C^2^ connection in **Int(B)** *cf. ortho*-carborane contrasts with the 2e addition to 1,2-Ph_2_-1,2-*closo*-C_2_B_10_H_10_ (towards [**7,9**-Ph_2_-**7,9**-*nido*-C_2_B_10_H_10_]^2–^), where the C^1^–C^2^ connection breaks due to a σ-C–C antibonding component in the LUMO orbital of the neutral species.^24^ In the LUMO of *ortho*-carborane (Fig. 9a) a π-antibonding interaction is seen between the C vertices, suggesting the connection would indeed be lengthened on occupation of the orbital. Further antibonding interactions are seen between C^2^ and the B^3^–B^7^ edge and those equivalent by *C*_2v_ symmetry (C^1^–{B^3^–B^4^}, C^1^–{B^5^–B^6^} and C^2^–{B^6^–B^11^}) and along the B^4^–B^5^ (and B^7^–B^11^) connections. Upon visualising the optimisation an initial lengthening of connections with antibonding interactions was noted (see ESI† for Movie). A snapshot of this (Fig. 8, **SS(*o*-B)**) shows the C^1^–C^2^ connection initially lengthens from 1.64 Å in *ortho*-carborane to *ca.* 1.7 Å. In addition, the distances from the C vertices to B^3^ and B^6^ and the B^4^–B^5^ and B^7^–B^11^ connections are also lengthened at **SS(*o*-B)**. As the optimisation continues the *C*_2v_ symmetry is reduced to *C*_2_ by reformation of the C^2^–B^3^ (1.55 Å), C^1^–B^5^ (1.71 Å) and C^1^–C^2^ (1.52 Å) connections in **Int(B)**.

**Fig. 10:**
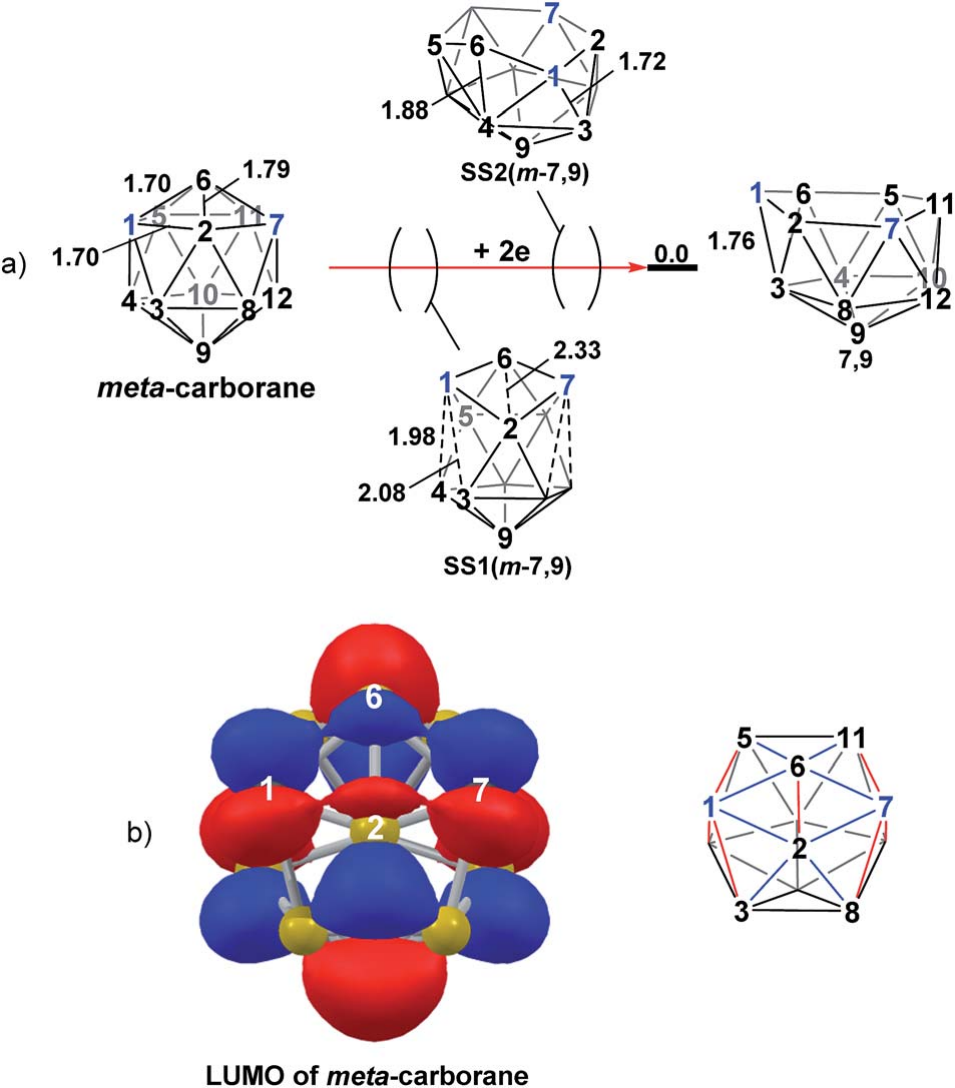
(a) 2e addition to *meta*-carborane giving **7,9**. Numbering of CH vertices (blue) and BH vertices (black) consistent with *meta*-carborane with selected distances in Å. (b) Left: representation of the LUMO orbital of *meta*-carborane at the BP86/6-31G** level; contour value = 0.05. Right: qualitative schematic shows bonding in blue and antibonding interactions in red. H atoms omitted for clarity.

Addition of 2e to *meta*-carborane led directly to the location of **7,9** (Fig. 10a) (see ESI† for movie). During the optimisation the structure initially distorts with retention of *C*_2v_ symmetry, consistent with the population of the LUMO of *meta*-carborane (Fig. 10b and see snap-shot geometry **SS1(*m*-7,9)** in Fig. 10a). Subsequently, B^6^–C^7^ lengthens and the symmetry is lost. At **SS2(*m*-7,9)**, a B^6^–B^4^ connection is formed and the connections from C^7^ to B^8^, B^11^ and B^12^ have reformed. **SS2(*m*-7,9)** is equivalent to **SS2(1,7-7,9)** (Fig. 4) and indeed undergoes a related DSD process, here in the B^3^–B^4^–B^6^–C^1^ diamond to give **7,9**.

## Discussion

Polyhedral expansion of *closo*-C_2_B_10_ carboranes with metal fragments produces a range of MC_2_B_10_ species which imply the intermediacy of **1,7**-, **3,7**-, **4,7**-, **7,9**- and **7,10**-isomers of the *nido*-[C_2_B_10_]^2–^ species. Here we have used DFT calculations to characterise the isomerisation pathways that link these various *nido* isomers. Our study has revealed several unusual new intermediates and their unforeseen rearrangement pathways which are categorised and rationalised below.

**Scheme 3:**
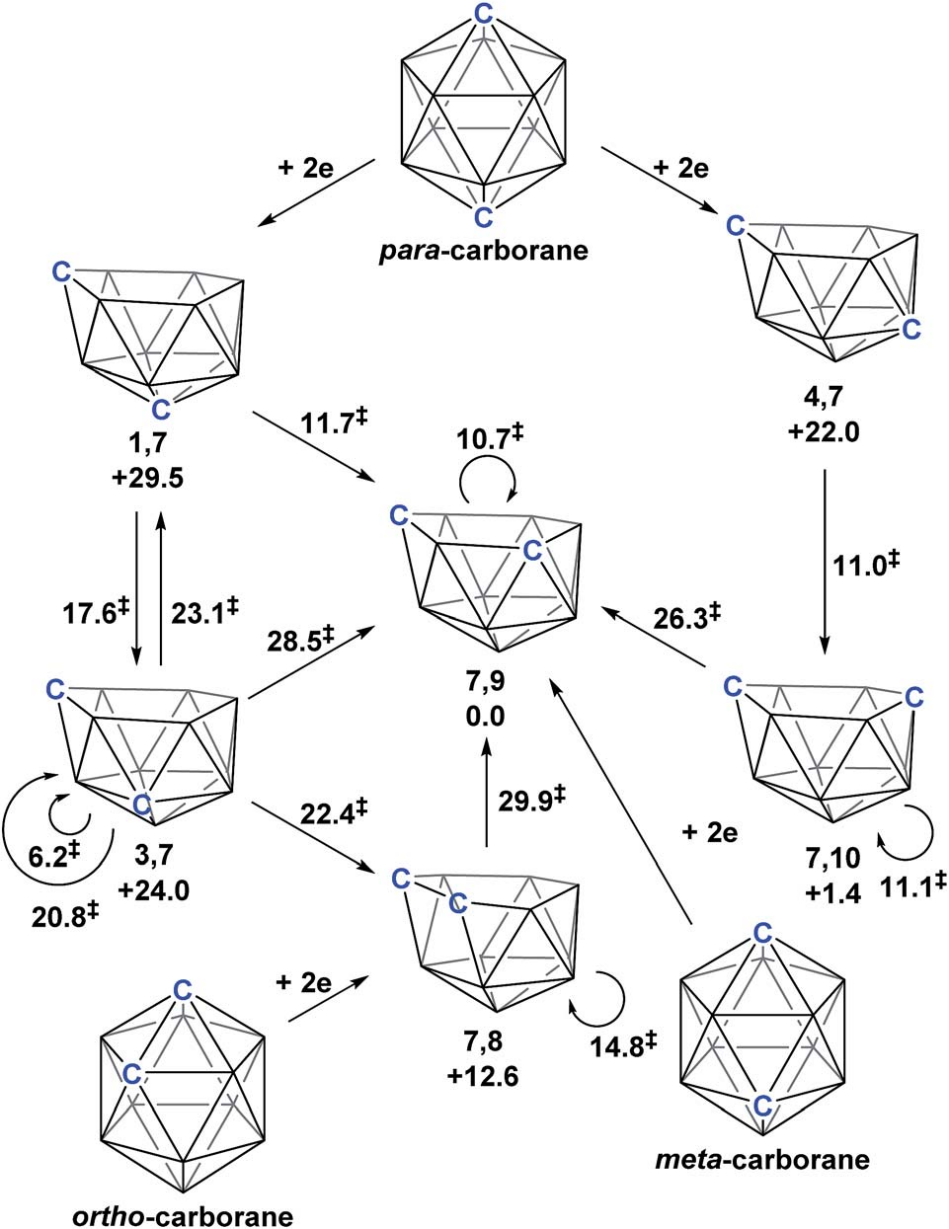
Interconnection of 12-vertex *nido* carborane dianions and their relation to 12-vertex *closo* carboranes. Relative energies of *nido* species and the barriers associated with rearrangement processes (denoted ^‡^) given in kcal mol^–1^.

Following the addition of two electrons to the optimised geometry of *para*-carborane, **1,7** and **4,7** are formed as the initial *nido* species (Scheme 3). Thereafter, **1,7** connects to **7,9**, through a single transition state, with a barrier of 11.7 kcal mol^–1^. In contrast, the isomerisation of **4,7** proceeds through a facile multi-step process to form **7,10**, but with a similar overall barrier of 11.0 kcal mol^–1^. The remaining *nido* species, **3,7**, is formed in an alternative 3-step process from **1,7** with a barrier of 17.6 kcal mol^–1^. In order to connect all *nido* species to the global minimum, **7,9**, additional pathways were sought from **7,10**, **3,7** and **4,7**. From **7,10**, a two-step process was characterised with a barrier of 26.3 kcal mol^–1^. From **3,7**, while a single step process was characterised for isomerisation to **7,9** with a barrier of 28.5 kcal mol^–1^ (see ESI Fig. S2†) this is higher than the reverse process from **3,7** to **1,7** (above; Δ*H* ^‡^ = 23.1 kcal mol^–1^) and therefore **3,7** likely isomerises to **7,9** *via* 1,7. Similarly, a direct pathway from **4,7** to **7,9** was not found and so formation of **7,9** from **4,7** is thought to proceed through **7,10** (Δ*H* ^‡^ = 11.0 kcal mol^–1^). An additional *nido* species, **7,8**, was found to be formed following 2e addition to *ortho*-carborane and isomerises to **7,9** through a barrier of 29.9 kcal mol^–1^. 2e reduction of *meta*-carborane leads directly to **7,9**. Degenerate pathways, where the start and end points of a rearrangement are the same *nido* species, were characterised for **7,9** (Δ*H* ^‡^ = 10.7 kcal mol^–1^), **7,10** (Δ*H* ^‡^ = 11.1 kcal mol^–1^), two examples for **3,7** (Δ*H* ^‡^ = 6.2 kcal mol^–1^ and 20.8 kcal mol^–1^) and **7,8** (Δ*H* ^‡^ = 14.8 kcal mol^–1^).

**Scheme 4:**
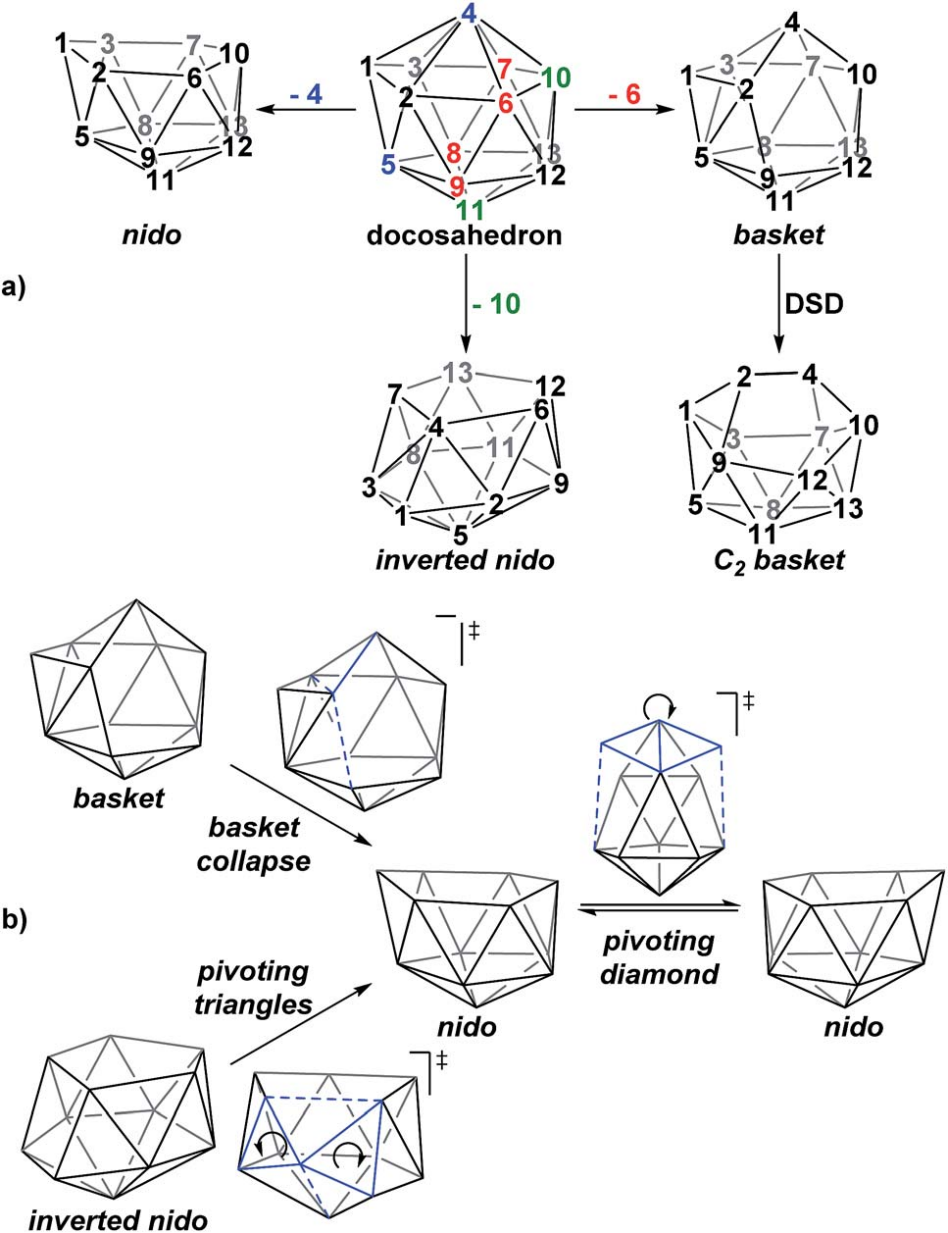
(a) Relationship between the docosahedron and *nido*, *basket* and *inverted nido* geometries with numbering consistent with the docosahedron. (b) Processes characterised that interconvert these geometries.

A family of *basket* intermediates, which are often energetically comparable to conventional *nido* fragments, were located along several of the characterised pathways. In a *basket* intermediate, two vertices form a basket handle bridging the remaining 10 vertices, with examples located in this study including **Int(A)**, **Int(A-4,7)**, **Int(7,10-7,9)**, **Int1(7,8-7,9)** and **Int2(7,8-7,9)** (all with *C*_1_ symmetry) and **Int2(4,7-7,10)** and **Int(B)** (with *C*_2_ symmetry with the CH vertices in the bridgehead positions and basket handle positions respectively). As shown in Scheme 4a, starting from the docosahedron, a *C*_1_-*basket* may be produced through removal of one of the 5-connected vertices 6–9 (red), with lengthening of the 1–4 distance to produce the requisite 4- and 5-membered faces. The *C*_2_-*basket* intermediates are related to the relevant *C*_1_-*basket* by a DSD in the 1–2–5–9 diamond and lengthening of the 3–4 distance. Two additional key intermediates, **Int1(4,7-7,10)** and **Int2(1,7-3,7)**, we refer to as *inverted nido* geometries, due to the 6-membered belt of vertices, rather than the 5-membered belt, being capped by a single vertex. The *inverted nido* motif is derived from the docosahedron by removal of a 5-connected vertex (10 or 11, green). A classical *nido* geometry is produced through removal of a 6-connected vertex (4 or 5, blue).^28^ In order to test the validity of this empirical observation, the idealised [B_13_H_13_]^2–^ docosahedron was computed, the appropriate vertices removed and the structure re-optimised as [B_12_H_12_]^4–^ fragments. The *nido* and *inverted nido* were located as minima (*H* = 0.0 and +25.8 respectively; see ESI†). The *basket* geometry was found to collapse to a *nido* structure, suggesting the C vertices are required to stabilise the distorted geometry, however, the *C*_2_ *basket* was located as a transition state that exchanges equivalent *nido* structures (*H* = +18.6 kcal mol^–1^; see ESI†).

The structural types discussed above tend to access specific rearrangement processes. From *nido* species, the initial step in the isomerisation is most often to move the 3-connected C^7^ vertex into a 4-connected position. This then triggers movement of the neighbouring vertices resulting in net rotation of the 6-membered belt of vertices above the 5-membered belt. Such processes are also responsible for the degenerate exchanges characterised in **7,8**, **7,9** and **7,10**. Three additional processes are found (Scheme 4b): a common DSD process by which *basket* intermediates undergo *basket collapse* to give *nido* species (Scheme 4b, upper left); the pivoting of a 4-vertex diamond about its long diagonal to directly exchange *nido* geometries (Scheme 4b, right; seen in the isomerisations from **1,7** to **7,9**, from **1,7** to **3,7** and in the higher energy degenerate process at **3,7** (see ESI, Fig. S1†)) and the pivoting of two triangles about a shared vertex exchanging *inverted nido* and *nido* geometries (Scheme 4b lower left; seen in the isomerisations of **4,7** to **7,10** and **1,7** to **3,7**).

## Conclusions

The rearrangements of dianionic 12-vertex *nido*-carboranes, [C_2_B_10_H_12_]^2–^, have been characterised through DFT calculations. *para*-Carborane rearranges to **1,7** and **4,7** as the first *nido* species, where experimentally, **1,7**, **3,7**, **4,7**, **7,9** and **7,10** are produced. Isomerisation processes have been characterised connecting **1,7** to **7,9**, **4,7** to **7,10**, **1,7** to **3,7** and **7,10** to **7,9**, thus rationalising the experimental formation of these species and showing how they are interconnected. Reduction of *ortho*-carborane gives **7,8** as the first *nido* species which subsequently isomerises to the expected **7,9**, while *meta*-carborane rearranges directly to **7,9**. The initial movements of vertices away from *closo* geometries, following the addition of two electrons, is related to the LUMO of the neutral species.

In the characterisation of these isomerisation processes, where possible through *a priori* mode-following calculations, a series of common intermediate topologies as well as the unexpectedly complex processes by which they interconnect have been uncovered and rationalised. *Basket* intermediates (*e.g.* **Int(A)**, **Int(A-4,7)**, **Int2(4,7-7,10)**, **Int(7,10-7,9)** and **Int(B)**) are characterised by a two-vertex basket handle bridging the remaining 10 vertices; *inverted nido* intermediates (**Int1(4,7-7,10)** and **Int2(1,7-3,7)**) exhibit a 5-membered belt and a 6-membered belt capped by the remaining vertex. The geometries of these new intermediates, like *nido* species themselves, are related to the 13-vertex docosahedron by removal of a single vertex. The pathways through which carborane dianions isomerise, driven by the thermodynamic preference for low-connected C vertices, are most often initiated by movement of the 3-connected C^7^ vertex, common to all *nido* species, into a 4-connected position through a DSD step, forcing a B vertex into a destabilised 3-connected site and leading to rearrangement of the cluster. Isomerisation continues through processes such as the pivoting of a 4-vertex diamond about its long diagonal. This can directly lead to a *nido* geometry or produce a *basket* or *inverted nido* intermediate. *Basket* intermediates can undergo a *basket collapse* process, characterised by DSD steps, giving rise to a *nido* geometry, while *inverted nido* intermediates convert to *nido* geometries through the pivoting of two 3-vertex triangles about a shared vertex.

## Computational details

Calculations were performed using Gaussian 03, Revision D.01 employing the BP86 functional^29,30^ and 6-31G** basis sets^31^ for B, C and H atoms. Zero-point corrected energies, *H*, are reported in kcal mol^–1^ relative to **7,9**. Analytical frequency calculations were used to confirm geometries as minima (all positive eigenvalues) or transition states (one negative eigenvalue). Transition states were further characterised through IRC calculations.^32,33^ Mode-following calculations used the ‘OPT = TS’ option in Gaussian along with the GDIIS algorithm, where convergence constraints were set to ‘verytight’ in order to force the optimisation to move away from a formally minimum energy starting geometry, itself optimised with default convergence constraints. Synchronous Transit-Guided Quasi-Newton (STQN) calculations were run with the ‘QST2’ option (two intermediate geometries given as input) and the structure generated used as input in a transition state optimisation.
