# Supplementary material for: Isomerisation of nido-[C2B10H12]2– dianions: unprecedented rearrangements and new structural motifs in carborane cluster chemistry
Source: Chem Sci. 2015 Mar 24;6(5):3117–28. doi: 10.1039/c5sc00726g (PMC5812468; doi:10.1039/c5sc00726g)
Supplement: Supplementary file 1 [file SC-006-C5SC00726G-s004.pdf]

Electronic Supplementary Material (ESI) for Chemical Science.
This journal is © The Royal Society of Chemistry 2015

SUPPORTING INFORMATION

Isomerisation of *nido*-$[C_2B_{10}H_{12}]^{2-}$ Dianions: Unprecedented Rearrangements and New Structural Motifs in Carborane Cluster Chemistry.

David McKay,* Stuart A. Macgregor and Alan J. Welch

Institute of Chemical Sciences, School of Engineering and Physical Sciences, Heriot-Watt University, Edinburgh, EH14 4AS, UK

Email: d.mckay@hw.ac.uk

Table of Contents

I. Additional isomerisation processes from 3,7....................2

II. $[B_{12}H_{12}]^{4-}$ *nido*, *inverted nido* and *basket* structures. 2

III. Computed geometries. 3

I. Additional isomerisation processes from 3,7.

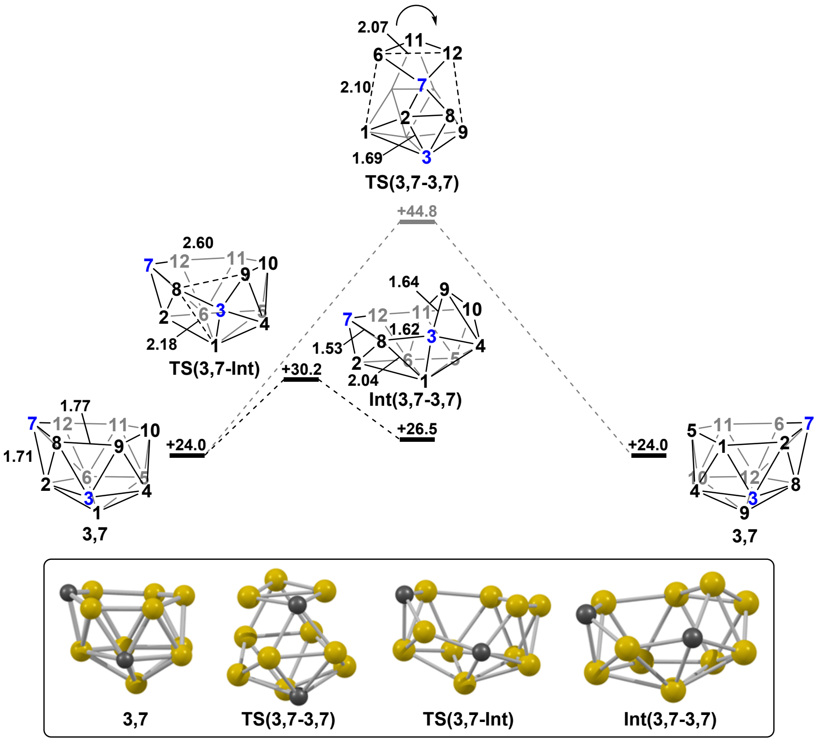

Figure S1. Degenerate pathways characterised from **3,7**. Numbering of CH vertices (blue) and BH vertices (black) consistent with **3,7** (left). Inset shows computed structures. Selected distances in Å and energies relative to **7,9** in kcal/mol. H atoms omitted for clarity.

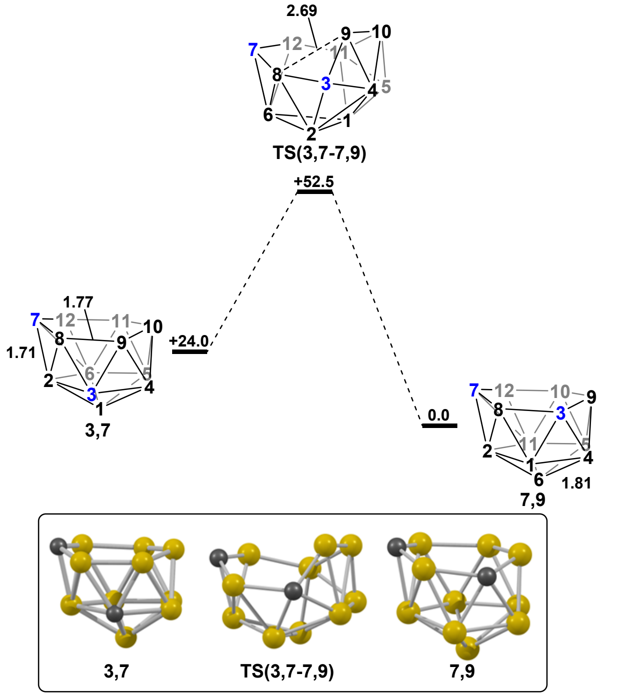

Figure S2. Formation of **7,9** from **3,7**. Numbering of CH vertices (blue) and BH vertices (black) consistent with **3,7** (left). Inset shows computed structures. Selected distances in Å and energies relative to **7,9** in kcal/mol. H atoms omitted for clarity.

II. *closo*-$[B_{13}H_{13}]^{2-}$, *nido*-$[B_{12}H_{12}]^{4-}$ and *inverted nido* and *C_2-basket* tetraanion structures.

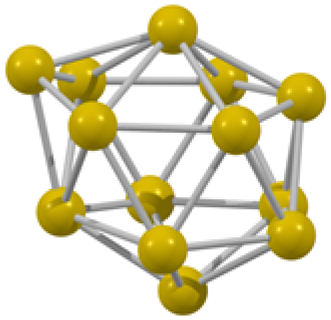

closo-$[B_{13}H_{13}]^{2-}$

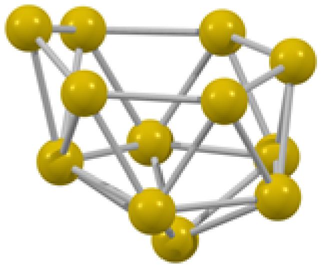

nido-$[B_{12}H_{12}]^{4-}$

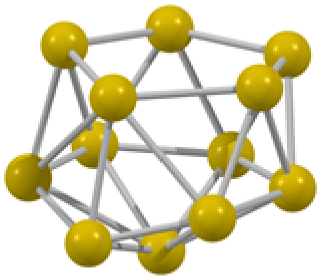

inverted nido

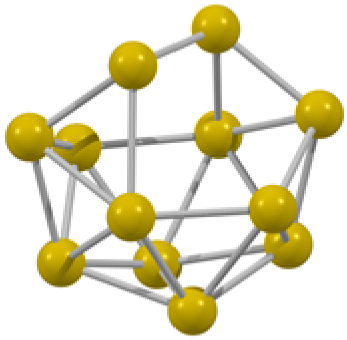

C_2-basket

III. Computed geometries.

***ortho*-carborane**

```
SCF Energy =              -332.070602164
Enthalpy 0K =             -331.898039
Enthalpy 298K =           -331.889084
Free Energy 298K =        -331.928934
```

Lowest Frequencies = 451.5 453.7 cm^{-1}

```
C   0.00017  0.81523 -1.28330
B   1.46116 -0.00169 -0.85677
B   1.45643  0.00259  0.91635
B   0.89595 -1.44453  0.03092
C   0.00090 -0.82102 -1.28049
B  -0.89511 -1.44526  0.03003
B  -0.00053 -0.89120  1.47152
B  -0.00091  0.89747  1.46660
B  -0.89590  1.44518  0.02404
B   0.89516  1.44549  0.02475
B  -1.46032 -0.00237 -0.85809
B  -1.45727  0.00180  0.91497
H   2.34660 -0.00317 -1.65348
H   1.49053 -2.46555 -0.13529
H   0.00135 -1.30444 -2.25934
H   2.49300  0.00383  1.51100
H  -1.48906 -2.46658 -0.13647
H   1.48969  2.46548 -0.14777
H  -0.00062 -1.53968  2.47468
H   0.00066  1.29398 -2.26426
H  -0.00140  1.55126  2.46743
H  -1.49103  2.46473 -0.14892
H  -2.34515 -0.00470 -1.65548
H  -2.49421  0.00212  1.50900
```

***meta*-carborane**

```
SCF Energy =              -332.095017031
Enthalpy 0K =             -331.921948
Enthalpy 298K =           -331.913073
Free Energy 298K =        -331.952799
```

Lowest Frequencies = 470.2 471.7 cm^{-1}

```
C   0.00005 -0.80480  1.30869
B  -0.00003  0.90875  1.43683
B   0.89930  1.46605  0.00056
B  -0.89943  1.46597  0.00058
B  -1.45125  0.01761  0.88934
B  -1.45126  0.01831 -0.88930
B  -0.00006  0.90987 -1.43612
B   1.45124  0.01844 -0.88932
B   0.89731 -1.41530 -0.00056
B   1.45126  0.01774  0.88931
B  -0.89718 -1.41538 -0.00054
C   0.00002 -0.80378 -1.30932
H  -0.00004  1.40885  2.52012
H  -1.54083  2.47405  0.00098
H  -2.40967 -0.08389  1.59194
H   1.54061  2.47419  0.00095
H  -2.40970 -0.08265 -1.59196
H   2.40971 -0.08367  1.59190
H  -0.00009  1.41082 -2.51902
H   0.00009 -1.39549  2.22727
H   2.40968 -0.08243 -1.59201
H   1.43462 -2.47736 -0.00098
H  -1.43440 -2.47749 -0.00096
H   0.00004 -1.39374 -2.22836
```

***para*-carborane**

```
SCF Energy =              -332.099505470
Enthalpy 0K =             -331.926232
Enthalpy 298K =           -331.917385
Free Energy 298K =        -331.957068
```

Lowest Frequencies = 462.1 467.3 cm^{-1}

```
C  -0.00910 -0.10732 -1.53184
B  -1.30158 -0.85028 -0.68656
B  -0.57464 -1.35360  0.85218
B  -1.51523  0.16798  0.75118
B  -1.16639  0.93187 -0.81222
B  -0.35580  1.53007  0.64871
C   0.00910  0.10732  1.53184
B   1.16639 -0.93187  0.81222
B   1.51523 -0.16798 -0.75118
B   0.35580 -1.53007 -0.64871
B   0.57464  1.35360 -0.85218
B   1.30158  0.85028  0.68656
H  -2.16494 -1.42323 -1.27628
H  -2.51879  0.28863  1.38345
H  -1.94017  1.54011 -1.48510
H  -0.95473 -2.24137  1.55135
H  -0.59085  2.55351  1.21330
H   0.59085 -2.55351 -1.21330
H   0.01556  0.18363  2.62153
H  -0.01556 -0.18363 -2.62153
H   1.94017 -1.54011  1.48510
H   2.51879 -0.28863 -1.38345
H   0.95473  2.24137 -1.55135
H   2.16494  1.42323  1.27628
```

1,7

```
SCF Energy =              -331.961143558
Enthalpy 0K =             -331.798629
Enthalpy 298K =           -331.788206
Free Energy 298K =        -331.830772
```

Lowest Frequencies = 198.4 326.4 cm^{-1}

```
C  -1.96764  0.00000 -0.53489
B  -1.14854 -0.00000  0.98116
C   0.55417 -0.00000  1.29100
B   0.04887  1.51154  0.75082
B   1.58134  0.88958  0.17822
B   1.58134 -0.88958  0.17822
B   0.04887 -1.51154  0.75082
B  -1.29481 -1.37724 -0.48308
B   0.42230 -1.54094 -0.95920
B   1.20712  0.00000 -1.36038
B   0.42230  1.54094 -0.95920
B  -1.29481  1.37724 -0.48308
H   0.71796  2.63299 -1.43234
H  -2.02909  2.36166 -0.40635
H  -3.07205  0.00000 -0.43719
H  -2.02909 -2.36166 -0.40636
H   0.71797 -2.63298 -1.43234
H   2.09719 -0.00000 -2.20689
H   0.80402 -0.00000  2.35719
H   0.00507 -2.38164  1.59845
H   2.58846 -1.41051  0.62235
```

H	2.58845	1.41051	0.62235
H	-1.78302	-0.00000	2.01457
H	0.00507	2.38164	1.59845

3,7

SCF Energy = -331.970132834
Enthalpy 0K = -331.807336
Enthalpy 298K = -331.796952
Free Energy 298K = -331.839478
Lowest Frequencies = 206.0 278.4 cm^{-1}

C	1.96819	-0.07418	-0.50443
B	1.14383	-0.09274	0.98885
B	-0.61378	0.00539	1.44352
C	-0.14597	-1.39375	0.64320
B	-1.62936	-0.78478	0.15643
B	-1.53773	0.98524	0.23575
B	0.04615	1.49399	0.80575
B	1.36789	1.33127	-0.50079
B	-0.34587	1.59884	-0.90307
B	-1.19982	0.12468	-1.35608
B	-0.49273	-1.45772	-1.01105
B	1.19588	-1.40947	-0.49202
H	-0.85418	-2.56365	-1.39241
H	1.82311	-2.45594	-0.35302
H	3.06777	-0.17198	-0.42645
H	2.15119	2.28040	-0.45484
H	-0.56558	2.71225	-1.36955
H	-2.07143	0.17215	-2.22075
H	-0.86337	-0.12811	2.62168
H	0.21456	2.40098	1.60113
H	-2.54666	1.58616	0.56390
H	-2.61400	-1.41224	0.49627
H	1.80076	-0.29578	1.98975
H	-0.14781	-2.29015	1.27526

4,7

SCF Energy = -331.973596651
Enthalpy 0K = -331.810574
Enthalpy 298K = -331.800256
Free Energy 298K = -331.842647
Lowest Frequencies = 197.4 340.1 cm^{-1}

B	-1.18074	0.04899	-1.36431
B	-0.39347	1.56252	-0.93020
B	1.32439	1.36623	-0.47790
C	1.97190	-0.01678	-0.53006
B	1.26741	-1.38129	-0.48197
B	-0.44303	-1.49168	-0.98684
C	-1.44796	-0.78305	0.17498
B	-1.54696	0.91827	0.21141
B	-0.01362	1.51624	0.78685
B	1.15552	-0.06077	1.00760
B	-0.05267	-1.50600	0.74258
B	-0.58952	0.01356	1.45748
H	-0.87868	-2.56108	-1.39434
H	1.97115	-2.38884	-0.42989
H	3.07742	-0.04633	-0.46531
H	2.07473	2.34057	-0.42008
H	-0.68566	2.64648	-1.42220
H	-2.16254	0.00942	-2.09964
H	-0.96721	-0.07964	2.60350
H	0.08619	2.46219	1.54535
H	-2.63118	1.37189	0.52380
H	-2.38696	-1.29054	0.43416
H	1.87218	-0.10262	1.98921
H	-0.14965	-2.49289	1.44233

7,8

SCF Energy = -331.989166729
Enthalpy 0K = -331.825521
Enthalpy 298K = -331.815400
Free Energy 298K = -331.857418
Lowest Frequencies = 217.7 340.6 cm^{-1}

C	1.68629	0.72651	-0.50543
C	1.68631	-0.72653	-0.50547
B	0.46808	-1.66731	-0.66887
B	-1.08417	-0.85935	-1.21696
B	-1.08411	0.85919	-1.21702
B	0.46813	1.66727	-0.66888
B	-0.89976	1.46315	0.44734
B	-1.85158	0.00003	0.16244
B	-0.89980	-1.46316	0.44752
B	0.77557	-0.98263	1.02177
B	-0.60771	-0.00002	1.46909
B	0.77544	0.98265	1.02165
H	0.77146	-2.84449	-0.83235
H	2.68876	-1.19484	-0.48975
H	2.68871	1.19487	-0.48939
H	0.77145	2.84452	-0.83190
H	-1.75991	1.53403	-1.98721
H	-1.76008	-1.53389	-1.98731
H	-0.86418	0.00031	2.65693
H	-1.41597	2.46903	0.90091
H	-3.04058	0.00010	0.43288
H	-1.41575	-2.46899	0.90148
H	1.39996	1.54371	1.90016
H	1.40002	-1.54337	1.90053

7,9

SCF Energy = -332.009355613
Enthalpy 0K = -331.845583
Enthalpy 298K = -331.835395
Free Energy 298K = -331.877620
Lowest Frequencies = 173.6 341.0 cm^{-1}

B	0.66249	0.09049	1.45877
B	0.22478	-1.46738	0.80603
B	-1.09716	-0.13817	1.05423
B	-0.08738	1.53010	0.77177
B	1.49234	1.06268	0.16542
B	1.66884	-0.69319	0.16920
B	0.22990	1.57871	-0.95146
B	-1.45310	1.23671	-0.42875
B	1.08607	0.12349	-1.36458
C	0.50252	-1.35106	-0.85721
B	-1.09547	-1.45465	-0.48257
C	-1.96468	-0.19774	-0.47320
H	0.37676	2.66843	-1.49342
H	-2.29129	2.13494	-0.35456
H	-3.05285	-0.36411	-0.36117
H	-1.61918	-2.56408	-0.50107
H	0.88692	-2.26898	-1.32979
H	1.88338	0.06459	-2.29427
H	0.96890	0.10291	2.63373

H 0.31822 -2.51135 1.41588
H 2.72769 -1.26705 0.33036
H 2.49242 1.71835 0.40261
H -1.80391 -0.24855 2.03673
H -0.27061 2.48378 1.50717

7,10

SCF Energy = -332.007184866
Enthalpy 0K = -331.843384
Enthalpy 298K = -331.833260
Free Energy 298K = -331.875278
Lowest Frequencies = 220.8 355.7 cm^{-1}
B -0.65113 0.00000 1.47032
B -0.05576 1.50967 0.77214
B 1.12790 -0.00000 1.03555
B -0.05576 -1.50966 0.77214
B -1.57943 -0.89035 0.20825
B -1.57943 0.89035 0.20825
B -0.38466 -1.45531 -0.96030
B 1.31430 -1.37222 -0.42448
C -1.09806 -0.00000 -1.22591
B -0.38466 1.45530 -0.96030
B 1.31430 1.37222 -0.42448
C 1.97872 0.00000 -0.50370
H -0.73629 -2.43642 -1.60177
H 2.03622 -2.36398 -0.36958
H 3.08557 0.00000 -0.46067
H 2.03622 2.36398 -0.36958
H -0.73630 2.43642 -1.60177
H -1.87307 -0.00000 -2.01089
H -0.95580 0.00000 2.64493
H -0.00263 2.51011 1.46103
H -2.65858 1.42591 0.36414
H -2.65858 -1.42591 0.36414
H 1.85362 -0.00000 2.01126
H -0.00262 -2.51011 1.46103

Int(A)

SCF Energy = -331.951946177
Enthalpy 0K = -331.790122
Enthalpy 298K = -331.779568
Free Energy 298K = -331.822415
Lowest Frequencies = 151.6 289.4 cm^{-1}
C -0.58361 -1.15726 -0.80968
B 0.93576 -1.37564 -0.13848
B 2.02272 0.00444 -0.20030
B 0.90633 -0.21503 1.34514
B -0.52662 -1.24538 0.92150
B -1.01115 0.32833 1.51102
C 1.38874 1.16544 0.57991
B 0.31048 1.54325 -0.56076
B -0.96154 0.50192 -1.25884
B 0.66658 -0.20910 -1.47939
B -1.82750 -0.35932 0.06310
B -1.40859 1.34068 0.20846
H 1.40519 -2.48168 -0.31201
H 1.49601 -0.60704 2.34302
H -0.74103 -2.31481 1.45468
H 3.18761 -0.36608 -0.34640
H -1.48011 0.30303 2.64070
H 0.95206 -0.49776 -2.62657

H 1.91814 1.92345 1.17768
H -0.95562 -2.00706 -1.39275
H 0.57341 2.48013 -1.31809
H -1.49924 0.67397 -2.33507
H -2.91405 -0.89355 -0.07141
H -2.30542 2.16754 0.10762

TS(A-1,7)

SCF Energy = -331.920420867
Enthalpy 0K = -331.759834
Enthalpy 298K = -331.749719
Free Energy 298K = -331.791697
Lowest Frequencies = -401.2 260.2 cm^{-1}
C 0.69363 -0.58847 1.22336
B -0.67774 -1.38397 0.63427
B -1.96247 -0.42502 -0.01594
B -0.59844 -1.07819 -1.16601
B 0.87456 -1.51028 -0.21163
B 0.93596 -0.29568 -1.52068
C -1.71104 0.89614 -0.64452
B -0.45343 1.74695 -0.15388
B 0.81257 1.09637 0.94734
B -0.77041 0.35414 1.28798
B 1.83296 -0.03660 0.01593
B 1.11168 1.34351 -0.79542
H -1.04940 -2.34218 1.27992
H -1.11626 -1.90432 -1.89942
H 1.38513 -2.60997 -0.09882
H -3.03734 -1.00450 0.05623
H 1.59132 -0.65215 -2.49467
H -1.25781 0.56026 2.37896
H -2.53173 1.42626 -1.18943
H 1.10139 -1.00194 2.15092
H -0.75751 2.90369 0.16402
H 1.27346 1.74095 1.86986
H 3.00755 -0.18712 0.29715
H 1.96949 2.16877 -1.09757

TS1(A-4,7)

SCF Energy = -331.944172932
Enthalpy 0K = -331.783166
Enthalpy 298K = -331.772927
Free Energy 298K = -331.815450
Lowest Frequencies = -178.3 107.6 cm^{-1}
C 0.76484 1.56307 0.05015
B -1.86838 0.80429 0.05910
B -1.17527 -0.28519 -1.16251
B -1.48903 -0.76738 0.58993
B -0.60798 0.69280 1.32141
B 0.12022 -0.99698 1.29649
C -0.22220 -1.48627 -0.32236
B 0.56891 -0.52195 -1.52396
B 1.75257 0.50170 -0.64641
B -0.59827 1.48767 -0.80252
B 1.24841 0.34268 1.08504
B 1.38904 -1.06814 0.01699
H -3.05763 1.07895 0.06401
H -2.33170 -1.54125 1.00335
H -0.77273 1.17920 2.42726
H -1.91187 -0.73818 -2.02051
H 0.22118 -1.82679 2.17339

H -0.73268 2.31604 -1.70842
H -0.40182 -2.54916 -0.52029
H 1.10075 2.55003 0.41412
H 0.79334 -1.17250 -2.53376
H 2.93734 0.69132 -0.88240
H 2.02157 0.59470 1.98812
H 2.17731 -1.99075 0.06063

Int(A-4,7)
SCF Energy = -331.944645640
Enthalpy 0K = -331.783515
Enthalpy 298K = -331.772460
Free Energy 298K = -331.816985
Lowest Frequencies = 47.6 214.4 cm^{-1}
C 0.51048 1.62500 -0.07073
B -2.00360 0.49310 0.02156
B -1.12191 -0.55604 -1.12602
B -1.32953 -0.88944 0.71376
B -0.63421 0.80821 1.16887
B 0.31037 -0.81893 1.37846
C -0.00743 -1.53700 -0.15560
B 0.62454 -0.65559 -1.49297
B 1.60313 0.64821 -0.78317
B -0.90714 1.38531 -0.80727
B 1.27451 0.59245 0.98677
B 1.54469 -0.88113 0.06556
H -3.21993 0.57704 0.03528
H -2.02972 -1.71521 1.26849
H -0.85574 1.41627 2.20092
H -1.82482 -1.21686 -1.87339
H 0.49685 -1.52041 2.34798
H -1.21074 2.22777 -1.65578
H -0.05697 -2.63062 -0.21572
H 0.75295 2.67607 0.16010
H 0.95445 -1.40664 -2.40014
H 2.69646 1.05479 -1.14630
H 2.01927 1.05506 1.82678
H 2.45545 -1.67606 0.18204

TS2(A-4,7)
SCF Energy = -331.944568273
Enthalpy 0K = -331.783591
Enthalpy 298K = -331.773342
Free Energy 298K = -331.815593
Lowest Frequencies = -85.6 221.3 cm^{-1}
C 0.47366 1.64340 -0.09570
B -2.01468 0.42516 0.00333
B -1.09135 -0.59417 -1.13946
B -1.30692 -0.92963 0.70714
B -0.66117 0.80290 1.15092
B 0.32481 -0.79434 1.38571
C 0.04022 -1.54579 -0.13595
B 0.65400 -0.67205 -1.48443
B 1.58230 0.67771 -0.79184
B -0.96049 1.39120 -0.78250
B 1.25047 0.63804 0.98283
B 1.56878 -0.84121 0.08150
H -3.23198 0.48786 0.00904
H -1.98643 -1.77041 1.26543
H -0.91158 1.41644 2.17258
H -1.78121 -1.28466 -1.87388

H 0.52115 -1.46991 2.37123
H -1.32279 2.25787 -1.58262
H 0.02233 -2.64148 -0.17643
H 0.70669 2.69950 0.12229
H 1.02315 -1.43119 -2.37012
H 2.66709 1.10861 -1.15417
H 1.97747 1.12761 1.82334
H 2.50407 -1.60399 0.21719

TS(1,7-7,9)
SCF Energy = -331.941151856
Enthalpy 0K = -331.779906
Enthalpy 298K = -331.769769
Free Energy 298K = -331.811843
Lowest Frequencies = -200.5 214.3 cm^{-1}
C -0.01576 1.72219 -0.17349
B 0.99695 0.97154 0.91621
C 0.47274 -0.56278 1.27661
B 1.76159 -0.39927 0.10780
B 0.54401 -1.66437 -0.05915
B -0.99103 -1.23143 0.69923
B -0.91997 0.65582 1.02754
B -1.40111 1.19488 -0.75536
B -2.02435 -0.15139 -0.11186
B -0.80095 -1.04967 -1.12405
B 0.90327 -0.60626 -1.44630
B 1.30196 1.01784 -0.85563
H 1.53037 -1.16299 -2.34065
H 2.21153 1.75318 -1.21978
H 0.01135 2.80808 0.01920
H -2.06766 2.02049 -1.38252
H -3.20885 -0.43604 -0.06918
H -1.34533 -1.87770 -1.84356
H 0.73272 -0.91027 2.28069
H -1.27069 1.14996 2.08223
H -1.47366 -2.07066 1.43582
H 0.89726 -2.80842 0.16465
H 1.50800 1.61754 1.80699
H 2.88119 -0.72803 0.45517

TS1(4,7-7,10)
SCF Energy = -331.954896105
Enthalpy 0K = -331.792967
Enthalpy 298K = -331.782966
Free Energy 298K = -331.824900
Lowest Frequencies = -174.8 194.7 cm^{-1}
B 0.79136 1.20193 -1.09678
B 1.16130 -0.49407 -1.33272
B -0.13511 -1.60119 -0.88015
C -1.56280 -0.90392 -0.39657
B -1.90621 0.57873 -0.66975
B -0.78301 1.72028 -0.32744
C 0.57964 1.39001 0.57509
B 1.78304 0.32913 0.13127
B 1.18892 -1.32258 0.27876
B -0.47220 -1.33777 0.86771
B -1.12653 0.39632 0.95988
B 0.58389 -0.05841 1.44620
H -1.10302 2.89602 -0.21660
H -3.06944 0.90928 -0.88363
H -2.42209 -1.59501 -0.31544

```
H  -0.26419 -2.75654 -1.27197
H   2.02693 -0.83357 -2.13461
H   1.34698  2.15366 -1.62912
H   0.88207  0.00003  2.61917
H   1.96365 -2.18949  0.64486
H   2.90860  0.65404  0.46230
H   0.89662  2.26525  1.15458
H  -0.88074 -2.13858  1.68744
H  -1.81369  0.65650  1.92700
```

Int1(4,7-7,10)

```
SCF Energy =                -331.961527507
Enthalpy 0K =               -331.799425
Enthalpy 298K =             -331.788855
Free Energy 298K =          -331.831685
```
Lowest Frequencies = 202.6 226.3 cm^{-1}
```
B   1.17467  0.03992 -1.18002
B  -0.03105 -1.31920 -1.25478
B  -1.56757 -0.73145 -0.76496
C  -1.51320  0.91029 -0.38405
B  -0.16813  1.55028 -0.92394
B   1.23140  1.43858 -0.00693
C   1.72616  0.05038  0.50116
B   1.11546 -1.39407  0.08979
B  -0.58157 -1.60633  0.44864
B  -1.38029 -0.11649  0.94357
B  -0.43702  1.38873  0.84759
B   0.34219 -0.34700  1.43996
H   2.02393  2.36480 -0.10106
H  -0.22526  2.61367 -1.53010
H  -2.44929  1.48298 -0.45316
H  -2.67901 -1.16915 -1.04195
H   0.13230 -2.28830 -1.98927
H   2.10530  0.09775 -1.96951
H   0.55928 -0.51227  2.62335
H  -0.92809 -2.67697  0.91406
H   1.90333 -2.30450  0.29464
H   2.74790 -0.03232  0.89336
H  -2.24027 -0.10698  1.80435
H  -0.71835  2.25249  1.65818
```

TS2(4,7-7,10)

```
SCF Energy =                -331.958281706
Enthalpy 0K =               -331.797022
Enthalpy 298K =             -331.786977
Free Energy 298K =          -331.828823
```
Lowest Frequencies = -255.2 260.6 cm^{-1}
```
B  -1.23992 -0.15683 -1.10551
B  -0.07380  1.23146 -1.28143
B   1.51097  0.76724 -0.76619
C   1.60849 -0.81983 -0.27042
B   0.28475 -1.28669 -1.04877
B  -1.07743 -1.55899 -0.00455
C  -1.67853 -0.21639  0.59136
B  -1.21471  1.28862  0.05517
B   0.45757  1.63532  0.40214
B   1.34867  0.19913  0.99079
B   0.50321 -1.39786  0.83830
B  -0.38152  0.40998  1.46290
H  -1.78442 -2.52773 -0.24627
H   0.44362 -2.11493 -1.93920
```

```
H   2.57591 -1.32421 -0.37791
H   2.57010  1.26013 -1.13058
H  -0.27629  2.10344 -2.11431
H  -2.15976 -0.28004 -1.89463
H  -0.58002  0.67519  2.63304
H   0.75579  2.75840  0.76465
H  -2.08101  2.13427  0.18986
H  -2.70764 -0.18739  0.97326
H   2.20563  0.29542  1.84851
H   0.86939 -2.23210  1.65364
```

Int2(4,7-7,10)

```
SCF Energy =                       -
331.981263472
Enthalpy 0K =              -331.818656
Enthalpy 298K =                        -
331.808292
Free Energy 298K =                     -
331.850826
Lowest Frequencies = 168.5179 217.8323
cm-1
B   1.24702  0.02387 -1.12448
B   0.21220 -1.43013 -0.88150
B  -1.41100 -1.01082 -0.36730
C  -1.66959  0.61629 -0.39871
B  -0.50036  0.14895 -1.51379
B   0.73157  1.65124 -0.43571
C   1.66959  0.61629  0.39871
B   1.41100 -1.01082  0.36730
B  -0.21220 -1.43013  0.88150
B  -1.24702  0.02387  1.12448
B  -0.73157  1.65124  0.43571
B   0.50036  0.14895  1.51379
H   1.26584  2.54169 -1.09966
H  -0.78923  0.20441 -2.69739
H  -2.72900  0.80887 -0.62243
H  -2.36176 -1.72156 -0.63313
H   0.38517 -2.45394 -1.51496
H   2.10170  0.00726 -1.98865
H   0.78923  0.20440  2.69739
H  -0.38517 -2.45394  1.51495
H   2.36176 -1.72157  0.63312
H   2.72900  0.80887  0.62243
H  -2.10170  0.00725  1.98865
H  -1.26584  2.54169  1.09967
```

TS3(4,7-7,10)

```
SCF Energy =               -331.975114257
Enthalpy 0K =              -331.812969
Enthalpy 298K =            -331.803058
Free Energy 298K =         -331.844746
```
Lowest Frequencies = -236.2 227.4 cm^{-1}
```
B  -1.13018  0.77221  0.98262
B  -0.52419 -0.85549  1.33601
B   1.08485 -1.16972  0.66640
C   1.84804  0.10876  0.04099
B   0.64486  0.59810  1.25350
B  -0.19584  1.84069 -0.10211
C  -1.35830  0.86726 -0.70759
B  -1.69502 -0.63541  0.00804
B  -0.33855 -1.69681 -0.24304
```

B 0.98346 -0.79002 -1.15007
B 1.33643 1.37891 -0.64574
B -0.69733 -0.44099 -1.50345
H -0.47794 3.00461 0.18161
H 1.12684 1.02438 2.28427
H 2.93057 -0.05461 0.16067
H 1.82498 -1.96327 1.21590
H -0.82943 -1.42762 2.36272
H -1.88102 1.31922 1.76441
H -1.22306 -0.81813 -2.54243
H -0.50597 -2.89920 -0.34173
H -2.84804 -1.01704 0.06407
H -2.30597 1.31805 -1.04214
H 1.72289 -1.41545 -1.89241
H 2.18540 2.06551 -1.22612

TS1(1,7-3,7)
SCF Energy = -331.933281850
Enthalpy 0K = -331.771307
Enthalpy 298K = -331.761550
Free Energy 298K = -331.802917
Lowest Frequencies = -154.0 287.0 cm^{-1}
C -0.64057 0.08731 1.28650
B 0.49239 -1.10694 0.99281
B -1.77087 0.37842 -0.00395
B -0.58150 1.60733 0.44472
B 0.87237 1.64624 -0.52025
B 0.93142 0.72932 1.01947
C 0.13413 -1.61421 -0.59437
B -1.20520 -1.23148 0.34692
B -1.01397 -0.60187 -1.31977
B -0.72081 1.11111 -1.25814
B 2.07106 0.44824 -0.52307
B 1.68074 -1.14231 -0.48394
H -0.99259 0.12154 2.32146
H 0.74319 -1.84345 1.91938
H -2.93195 0.57484 0.30383
H -1.01080 2.56438 1.06182
H 1.24992 2.81563 -0.54034
H 1.45627 1.04706 2.06943
H 0.04530 -2.70487 -0.73882
H -1.92380 -2.07692 0.83514
H -1.75505 -1.15509 -2.12282
H -1.29933 1.89181 -2.00662
H 3.23549 0.84287 -0.37911
H 2.44384 -2.10667 -0.35015

Int1(1,7-3,7)
SCF Energy = -331.936090701
Enthalpy 0K = -331.773889
Enthalpy 298K = -331.763476
Free Energy 298K = -331.805996
Lowest Frequencies = 227.6 257.2 cm^{-1}
C 0.61897 -0.21229 1.28531
B 0.33237 1.36665 0.84449
B 1.33171 -1.21923 0.06610
B -0.28163 -1.53870 0.68177
B -1.65535 -1.07663 -0.30375
B -1.11060 0.18618 0.99226
C 0.64492 1.34246 -0.81556
B 1.72190 0.47949 0.15369

B 1.06710 -0.16348 -1.39662
B -0.02147 -1.48412 -1.06712
B -2.09912 0.47658 -0.60556
B -0.94043 1.64386 -0.38203
H 0.94694 -0.32393 2.32260
H 0.62527 2.23561 1.63500
H 2.23729 -1.96016 0.40197
H -0.39811 -2.45266 1.47628
H -2.49397 -1.95897 -0.12861
H -1.65509 0.32804 2.07248
H 1.17377 2.22866 -1.20280
H 2.81474 0.87340 0.49939
H 1.91365 -0.17817 -2.28218
H 0.02152 -2.55183 -1.66722
H -3.29473 0.78641 -0.62496
H -1.19698 2.83959 -0.23657

TS2(1,7-3,7)
SCF Energy = -331.931795097
Enthalpy 0K = -331.770578
Enthalpy 298K = -331.760536
Free Energy 298K = -331.802439
Lowest Frequencies = -213.7 216.2 cm^{-1}
C 0.70733 -0.54694 1.23583
B 0.72562 1.08882 0.96270
B 0.88809 -1.51985 -0.13804
B -0.67386 -1.39281 0.68699
B -1.96259 -0.56197 -0.06465
B -1.04471 0.49249 1.04889
C 1.01650 1.19424 -0.72906
B 1.81448 -0.06596 0.05654
B 0.89849 -0.29761 -1.46862
B -0.60535 -1.14578 -1.14574
B -1.81285 0.95268 -0.65256
B -0.41212 1.79614 -0.20248
H 1.08662 -0.88568 2.20403
H 1.27235 1.79046 1.78725
H 1.47324 -2.57876 -0.01277
H -0.96503 -2.35262 1.37767
H -3.00496 -1.18117 0.09192
H -1.45729 0.75618 2.16875
H 1.77680 1.92228 -1.05468
H 3.00171 -0.05015 0.30183
H 1.61046 -0.51518 -2.43995
H -0.96201 -2.05979 -1.87907
H -2.81681 1.54263 -1.06476
H -0.43404 2.99721 0.06405

Int2(1,7-3,7)
SCF Energy = -331.959765540
Enthalpy 0K = -331.798110
Enthalpy 298K = -331.787513
Free Energy 298K = -331.830327
Lowest Frequencies = 203. 8 265.4 cm^{-1}
C -0.62493 -1.06499 -1.05996
B -0.71321 0.61546 -1.27330
B -0.37137 -1.58568 0.55678
B 0.91310 -1.48779 -0.66586
B 1.95020 -0.07658 -0.43856
B 1.02355 1.27194 -0.94717
C -1.57981 0.92050 0.25775

B -1.74108 -0.61367 0.15630
B -0.46906 0.11879 1.38142
B 1.14357 -0.78942 0.98304
B 1.20606 0.96075 0.81111
B -0.24528 1.75850 0.13166
H -1.15776 -1.74477 -1.74427
H -1.37357 1.00110 -2.22688
H -0.73818 -2.66948 0.96052
H 1.32296 -2.60149 -0.97489
H 3.17458 -0.14486 -0.47314
H 1.50758 2.29796 -1.41884
H -2.45218 1.51549 0.55339
H -2.83865 -1.12570 0.30376
H -0.79938 0.21714 2.54349
H 1.72616 -1.30985 1.92141
H 1.83096 1.61254 1.63352
H -0.45648 2.95744 0.25808

TS3(1,7-3,7)
SCF Energy = -331.949165653
Enthalpy 0K = -331.788344
Enthalpy 298K = -331.778116
Free Energy 298K = -331.820308
Lowest Frequencies = -296.9 254.8 cm^{-1}
C 1.20247 -0.97194 -0.87853
B -0.84235 -0.68588 -1.23923
B 1.42600 -0.67779 0.75837
B 1.75214 0.48473 -0.59235
B 0.52922 1.78678 -0.59563
B -0.96590 1.09385 -1.12071
C -1.29568 -1.24603 0.28330
B 0.16605 -1.76724 0.05335
B -0.34659 -0.25707 1.37756
B 0.96496 1.00653 0.95578
B -0.70591 1.48630 0.62450
B -1.83891 0.22659 0.18621
H 1.86519 -1.72180 -1.34190
H -1.25659 -1.36181 -2.16415
H 2.23905 -1.25596 1.45246
H 2.93999 0.69448 -0.81633
H 0.86292 2.95782 -0.74998
H -1.78997 1.74156 -1.76495
H -1.97715 -2.03465 0.61906
H 0.29204 -2.97893 0.14552
H -0.58349 -0.49012 2.54367
H 1.52412 1.56071 1.89049
H -1.21652 2.37719 1.28314
H -3.03389 0.33539 0.43512

TS1(7,10-7,9)
SCF Energy = -331.963101809
Enthalpy 0K = -331.801449
Enthalpy 298K = -331.791543
Free Energy 298K = -331.833164
Lowest Frequencies = -377.3 273.7 cm^{-1}
B 0.84573 -0.71384 1.31937
B 0.90362 -1.46998 -0.27796
B -0.61696 -1.43469 0.61395
B -0.70260 0.22815 1.40369
B 0.81011 1.06067 1.02758
B 1.83574 0.02121 0.02212

B -0.52855 1.71906 -0.01113
B -1.96424 -0.49870 0.03073
B 0.99030 1.37087 -0.74943
C 0.83544 -0.16117 -1.39015
B -0.59330 -0.91449 -1.17875
C -1.74605 0.83437 -0.56844
H -0.87457 2.84786 0.34842
H -3.01078 -1.13540 0.02589
H -2.55193 1.34258 -1.15003
H -1.00552 -1.65310 -2.05714
H 1.37831 -0.39000 -2.32038
H 1.81389 2.15584 -1.20307
H 1.35513 -1.24846 2.28386
H 1.45405 -2.52156 -0.53549
H 3.04657 -0.06945 -0.00843
H 1.27893 1.82109 1.85387
H -1.01966 -2.48405 1.07690
H -1.30007 0.45413 2.43634

Int(7,10-7,9)
SCF Energy = -332.004225647
Enthalpy 0K = -331.841043
Enthalpy 298K = -331.830919
Free Energy 298K = -331.872855
Lowest Frequencies = 268.8 294.3 cm^{-1}
B -0.71244 1.42209 -0.66861
B -1.04503 -0.24022 -1.28095
B 0.56594 0.45602 -1.47615
B 0.92566 1.42616 -0.01444
B -0.46557 1.14542 1.10857
B -1.80563 0.35030 0.24341
B 0.99434 0.06272 1.29328
B 1.99758 0.06264 -0.29957
B -0.78956 -0.54273 1.44411
C -1.26023 -1.22519 0.06492
B 0.22531 -1.43710 -0.75136
C 1.40707 -1.22837 0.29623
H 1.64551 0.26217 2.30832
H 3.16992 0.40915 -0.41558
H 1.98266 -2.05358 0.74919
H 0.30306 -2.32614 -1.59818
H -2.05624 -1.98013 -0.00089
H -1.21213 -0.91994 2.52395
H -1.18928 2.43982 -1.13323
H -1.71451 -0.47961 -2.26614
H -2.99080 0.59078 0.35614
H -0.64308 2.01053 1.94512
H 0.87204 0.74176 -2.62089
H 1.49889 2.50007 -0.00611

TS2(7,10-7,9)
SCF Energy = -331.973457189
Enthalpy 0K = -331.811445
Enthalpy 298K = -331.801604
Free Energy 298K = -331.843125
Lowest Frequencies = -402.3 265.7 cm^{-1}
B -0.77175 -0.73002 1.33221
B -0.86342 1.00748 1.01552
B 0.72886 0.30823 1.34708
B 0.72310 -1.40193 0.61342
B -0.82342 -1.55113 -0.24936

B -1.82823 -0.14224 0.01645
B 0.65994 -1.02537 -1.18093
B 1.98404 -0.38059 0.01269
B -0.85633 -0.24898 -1.50922
C -1.04749 1.18964 -0.68886
B 0.36502 1.71710 -0.11785
C 1.69193 0.96034 -0.55882
H 1.21579 -1.77698 -1.96614
H 3.10102 -0.87345 0.09533
H 2.52493 1.57394 -0.98684
H 0.48314 2.92334 0.10477
H -1.80720 1.92565 -0.99668
H -1.50492 -0.41737 -2.53368
H -1.21272 -1.22338 2.34997
H -1.40935 1.78329 1.77317
H -3.04120 -0.14824 0.08412
H -1.29709 -2.66744 -0.36318
H 1.28819 0.62249 2.37736
H 1.20374 -2.38445 1.14785

Int(B)
SCF Energy = -331.979297522
Enthalpy 0K = -331.816260
Enthalpy 298K = -331.806040
Free Energy 298K = -331.848261
Lowest Frequencies = 179.4 282.0 cm^{-1}
C 0.61115 -1.52740 0.45416
B -1.82739 -0.74682 0.11027
B -1.47864 0.98717 0.02204
B -0.85957 -0.09386 -1.34721
C -0.61115 -1.52740 -0.45416
B 0.97240 0.11121 -1.48113
B 0.00312 1.43530 -0.90566
B -0.00312 1.43530 0.90567
B 0.85957 -0.09386 1.34721
B -0.97239 0.11121 1.48113
B 1.82738 -0.74682 -0.11027
B 1.47864 0.98717 -0.02204
H -3.00389 -1.02955 -0.06541
H -1.46967 -0.16904 -2.39926
H -0.66529 -2.40765 -1.12590
H -2.44441 1.72672 -0.02122
H 1.45861 0.13192 -2.59884
H -1.45861 0.13192 2.59884
H -0.12590 2.44705 -1.56929
H 0.66528 -2.40765 1.12590
H 0.12591 2.44705 1.56930
H 1.46967 -0.16904 2.39926
H 3.00389 -1.02956 0.06541
H 2.44441 1.72672 0.02123

TS(B-7,9)
SCF Energy = -331.921718590
Enthalpy 0K = -331.761328
Enthalpy 298K = -331.751185
Free Energy 298K = -331.793222
Lowest Frequencies = -484.5 224.5 cm^{-1}
C -1.53287 1.13986 -0.49554
B 0.70205 -0.10984 -1.56718
B 1.81087 -0.26421 -0.13233
B 0.63782 -1.54218 -0.47189

C -0.77890 -0.72181 -1.07491
B -0.78644 -1.34400 0.59748
B 0.79968 -0.95324 1.20406
B 1.03112 0.83668 1.05370
B -0.23045 1.76571 0.13889
B 1.23782 1.30906 -0.67512
B -1.96709 -0.14582 0.12330
B -0.55403 0.17328 1.52334
H 1.01751 -0.41634 -2.71010
H 0.83191 -2.69225 -0.81446
H -1.35487 -1.28426 -1.82222
H 2.99403 -0.55277 -0.16644
H -1.38176 -2.35668 0.89784
H 2.14711 2.07853 -0.96288
H 1.28082 -1.66558 2.06302
H -2.15122 1.70409 -1.23515
H 1.63997 1.42204 1.93323
H -0.43919 2.90849 0.56340
H -3.08940 -0.63727 0.07965
H -1.03099 0.35650 2.62561

TS(B-7,8)
SCF Energy = -331.953085554
Enthalpy 0K = -331.791556
Enthalpy 298K = -331.781501
Free Energy 298K = -331.823494
Lowest Frequencies = -394.7 186.1 cm^{-1}
C -0.14266 1.75314 -0.09724
B 1.26507 1.27906 -0.59385
B 1.77445 -0.23223 0.22535
B 1.10268 -0.30792 -1.43100
C -1.40770 1.12875 -0.60252
B -0.53706 -0.88338 -1.32702
B 0.77020 -1.59753 -0.26247
B 0.51695 -0.85454 1.34471
B -0.96379 0.05158 1.36581
B 0.66789 0.92053 1.14906
B -1.92615 -0.21274 -0.22725
B -0.88890 -1.45304 0.39954
H 2.17300 2.10065 -0.68072
H 1.83681 -0.58970 -2.37093
H -2.06904 1.84213 -1.15536
H 2.94657 -0.38678 0.52053
H -1.08105 -1.48924 -2.23678
H 1.07633 1.57386 2.09243
H 1.17169 -2.74478 -0.35936
H -0.28555 2.82046 0.16096
H 0.85802 -1.35137 2.39961
H -1.63941 0.36053 2.32346
H -3.08975 -0.52000 -0.42231
H -1.50209 -2.45601 0.71256

TS1(7,8-7,9)
SCF Energy = -331.963691133
Enthalpy 0K = -331.801888
Enthalpy 298K = -331.791809
Free Energy 298K = -331.833908
Lowest Frequencies = -74.9 166.8 cm^{-1}
C 0.77972 1.58332 -0.00572
C 1.64620 0.48344 -0.53970
B 0.80054 -0.59520 -1.42823

B -0.98675 -0.35535 -1.30379
B -1.93546 0.63250 -0.14349
B -0.68224 1.56898 -0.67397
B -0.92081 0.58412 1.26000
B -1.53831 -0.92194 0.40533
B -0.10555 -1.67776 -0.33835
B 1.40934 -1.04315 0.22671
B 0.01672 -0.95795 1.31792
B 1.02946 0.49329 1.19080
H 1.34379 -1.12389 -2.38790
H 2.72936 0.67170 -0.63642
H 1.27018 2.54911 0.20827
H -1.00110 2.56700 -1.33533
H -3.09423 0.96978 -0.30669
H -1.63886 -0.72199 -2.27300
H 0.10185 -1.58458 2.35596
H -1.23450 1.13920 2.29830
H -2.42884 -1.67522 0.75943
H -0.20836 -2.87453 -0.54383
H 1.78084 0.76771 2.10753
H 2.38965 -1.72256 0.46150

Int1(7,8-7,9)
SCF Energy = -331.963929768
Enthalpy 0K = -331.801912
Enthalpy 298K = -331.791148
Free Energy 298K = -331.834865
Lowest Frequencies = 92.1 145.7 cm^{-1}
C -0.51712 -1.58281 0.11174
C -1.53311 -0.67690 -0.55862
B -0.68795 0.35835 -1.51984
B 1.09469 0.35947 -1.21835
B 1.99726 -0.51148 0.08445
B 0.89569 -1.47550 -0.67245
B 0.72095 -0.48547 1.34006
B 1.38258 1.02468 0.49824
B 0.00277 1.63899 -0.46775
B -1.51817 0.93641 0.00720
B -0.25198 1.08666 1.23643
B -1.13227 -0.42622 1.14404
H -1.16550 0.69488 -2.59261
H -2.55610 -1.04355 -0.73192
H -0.86032 -2.56691 0.47693
H 1.17121 -2.41680 -1.42561
H 3.21160 -0.62562 0.11609
H 1.78960 0.74319 -2.14840
H -0.45888 1.81972 2.18309
H 0.93822 -0.96242 2.44059
H 2.17676 1.84457 0.92629
H 0.04071 2.81768 -0.77209
H -1.92106 -0.79562 1.98869
H -2.58273 1.51974 0.06011

TS2(7,8-7,9)
SCF Energy = -331.938593313
Enthalpy 0K = -331.777905
Enthalpy 298K = -331.767785
Free Energy 298K = -331.809793
Lowest Frequencies = -516.2 192.8 cm^{-1}
C 1.49554 -0.94177 0.46009
B -1.12255 -1.30850 0.68375
B -1.09155 0.39863 1.21507
B -2.03414 -0.19549 -0.22277
C -0.15904 -1.46420 -0.56848
B -0.61791 -0.08376 -1.48760
B -1.09844 1.27503 -0.38414
B 0.28774 1.46559 0.76329
B 1.76330 0.61103 0.19804
B 0.55862 -0.21660 1.54498
B 1.12911 -0.48589 -1.10157
B 0.57907 1.22058 -0.98979
H -1.42636 -2.25629 1.39674
H -3.22108 -0.02359 -0.46565
H -0.10780 -2.41738 -1.12941
H -1.75225 0.76678 2.17183
H -0.79217 -0.18738 -2.68913
H 0.91340 -0.23763 2.71522
H -1.72697 2.25806 -0.73050
H 2.27463 -1.69847 0.60331
H 0.41619 2.55342 1.29580
H 2.88066 1.10410 0.15762
H 1.80393 -0.91543 -2.01606
H 0.95253 2.08646 -1.75573

Int2(7,8-7,9)
See Int(7,10-7,9)

TS3(7,8-7,9)
See TS2(7,10-7,9)

TS(3,7-3,7)
SCF Energy = -331.934314739
Enthalpy 0K = -331.774163
Enthalpy 298K = -331.763739
Free Energy 298K = -331.806281
Lowest Frequencies = -230.5 213.9 cm^{-1}
C -0.78549 -1.50316 -0.00008
B -1.51528 -0.36426 0.90431
B -0.29219 0.74078 1.45378
C -1.22407 1.03317 0.00025
B 0.35322 1.64692 -0.00018
B 1.41311 0.54327 0.90033
B 0.52643 -1.14696 1.03725
B 0.52619 -1.14722 -1.03708
B 1.87990 -0.86794 -0.00005
B 1.41243 0.54274 -0.90104
B -0.29303 0.74076 -1.45382
B -1.51607 -0.36409 -0.90378
H -0.52048 1.45522 -2.41282
H -2.60070 -0.52899 -1.42906
H -1.17574 -2.52738 -0.00006
H 0.53727 -1.82880 -2.05382
H 3.04613 -1.22826 -0.00048
H 2.22514 1.04587 -1.65797
H -0.51948 1.45495 2.41302
H 0.53756 -1.82845 2.05405
H 2.22604 1.04697 1.65665
H 0.42742 2.86000 -0.00025
H -2.59937 -0.52936 1.43062
H -1.99996 1.80821 0.00052

TS(3,7-Int)

SCF Energy = -331.959217340
Enthalpy 0K = -331.797429
Enthalpy 298K = -331.787258
Free Energy 298K = -331.829513
Lowest Frequencies = -103.8 236.0 cm^{-1}

C	1.79925	-0.55661	-0.87596
B	1.72743	-0.08437	0.71647
B	-0.09406	-0.35979	1.30971
C	-0.66664	-1.54460	0.16480
B	-1.79166	-0.37938	0.53903
B	-1.09535	1.20391	0.70027
B	0.66702	1.27707	0.81579
B	1.52304	0.99315	-0.74842
B	-0.16477	1.62845	-0.74633
B	-1.69390	0.68590	-0.89585
B	-1.38261	-0.93656	-1.11888
B	0.94783	-1.58749	-0.15495
H	-1.82470	-1.70661	-1.95300
H	1.39861	-2.70041	0.09161
H	2.81056	-0.89122	-1.17113
H	2.43579	1.76326	-1.03485
H	-0.26512	2.77876	-1.15756
H	-2.66939	1.25604	-1.36470
H	-0.14107	-0.71144	2.47070
H	1.01557	2.11468	1.63687
H	-1.70904	1.98262	1.40882
H	-2.75319	-0.77848	1.16984
H	2.63887	-0.17852	1.51826
H	-0.94740	-2.52590	0.56792

Int(3,7-3,7)

SCF Energy = -331.965582254
Enthalpy 0K = -331.803330
Enthalpy 298K = -331.792679
Free Energy 298K = -331.835683
Lowest Frequencies = 224.8 261.8 cm^{-1}

C	-2.03395	0.16919	-0.79382
B	-1.73882	-0.31946	0.75367
B	-0.06413	0.48298	1.25492
C	0.22436	1.50340	-0.16067
B	1.65128	0.91700	0.45281
B	1.33891	-0.81692	0.91822
B	-0.33304	-1.30593	0.79207
B	-1.21704	-1.16727	-0.78742
B	0.65586	-1.25495	-0.76712
B	2.15723	-0.38729	-0.51726
B	1.05772	0.62579	-1.26200
B	-1.38025	1.33870	-0.05301
H	1.26493	1.13139	-2.35372
H	-1.99378	2.31812	0.35721
H	-3.10654	0.18208	-1.04707
H	-1.74748	-2.19997	-1.17800
H	0.94606	-2.29563	-1.33709
H	3.26862	-0.76429	-0.82543
H	-0.07345	0.96541	2.36907
H	-0.45773	-2.26977	1.53251
H	1.93467	-1.41076	1.79708
H	2.39091	1.71406	1.00345
H	-2.61210	-0.55802	1.56841
H	0.40473	2.58862	-0.08385

TS(3,7-7,9)

SCF Energy = -331.922109206
Enthalpy 0K = -331.761881
Enthalpy 298K = -331.751035
Free Energy 298K = -331.794717
Lowest Frequencies = -163.1 169.0 cm^{-1}

C	1.85607	0.38615	-0.86157
B	1.50865	0.45797	0.77999
B	0.51455	-0.90648	1.17447
C	-0.01205	-1.72086	-0.16027
B	-1.29522	-0.85857	0.62436
B	-1.80391	0.83808	0.54813
B	-0.28224	0.72892	1.31193
B	0.97660	1.63153	-0.75410
B	-0.66209	1.71591	-0.29888
B	-1.71096	0.26106	-1.05533
B	-0.94441	-1.19669	-1.28766
B	1.55293	-1.03530	-0.30160
H	-1.35275	-2.06582	-2.05735
H	2.41608	-1.90024	-0.27416
H	2.92688	0.55823	-1.07725
H	1.57506	2.70631	-0.88533
H	-1.08718	2.86903	-0.24462
H	-2.74238	0.44839	-1.70582
H	0.69464	-1.54460	2.19088
H	-0.29724	1.11747	2.46861
H	-2.79881	1.26317	1.10268
H	-2.00127	-1.61542	1.26983
H	2.30820	0.79808	1.63106
H	0.02505	-2.80847	0.00601

***closo*-[$B_{13}H_{13}$]$^{2-}$**

SCF Energy = -331.037636224
Enthalpy 0K = -330.864230
Enthalpy 298K = -330.853068
Free Energy 298K = -330.897086
Lowest Frequencies = 136.6 231.1 cm^{-1}

B	2.11967	-0.00002	0.00000
B	1.28200	-1.40077	0.00000
B	1.28202	1.40076	0.00000
B	0.84796	-0.00001	-1.35120
B	0.84796	-0.00001	1.35120
B	-0.26945	-1.51102	-0.88396
B	-0.26943	1.51102	-0.88396
B	-0.26943	1.51102	0.88396
B	-0.26945	-1.51102	0.88396
B	-0.99001	0.00001	-1.43199
B	-0.99001	0.00001	1.43200
B	-1.65592	-0.89166	0.00000
B	-1.65590	0.89167	0.00000
H	3.33424	-0.00003	0.00000
H	1.92290	-2.43933	0.00000
H	1.92293	2.43930	0.00000
H	1.36105	-0.00001	-2.45318
H	1.36105	-0.00001	2.45318
H	-0.35484	-2.51158	-1.56631
H	-0.35481	2.51158	-1.56631
H	-0.35481	2.51158	1.56631
H	-0.35484	-2.51158	1.56631
H	-1.54647	0.00001	-2.51095
H	-1.54647	0.00001	2.51095

H -2.71998 -1.47566 0.00000
H -2.71997 1.47569 0.00000

***nido*-$[B_{12}H_{12}]^{4-}$**

SCF Energy = -304.815809702
Enthalpy 0K = -304.669406
Enthalpy 298K = -304.658330
Free Energy 298K = -304.701938
Lowest Frequencies = 172.9 314.4 cm^{-1}
B 2.18089 0.00000 -0.61609
B 1.34769 -1.43233 -0.43239
B 1.34769 1.43233 -0.43239
B 1.16249 0.00000 0.97036
B -0.40119 -1.54151 -0.98738
B -0.40119 1.54151 -0.98738
B -0.03359 1.52126 0.75901
B -0.03359 -1.52127 0.75901
B -1.23583 0.00000 -1.37119
B -0.62590 0.00000 1.45566
B -1.58777 -0.89430 0.19349
B -1.58777 0.89431 0.19350
H 3.46308 -0.00000 -0.58539
H 2.00102 -2.51737 -0.27710
H 2.00102 2.51737 -0.27710
H 1.85422 -0.00000 2.00976
H -0.75918 -2.65157 -1.48054
H -0.75917 2.65157 -1.48054
H -0.01618 2.49818 1.53788
H -0.01619 -2.49818 1.53788
H -2.19035 0.00000 -2.20696
H -0.94354 0.00000 2.65814
H -2.64715 -1.47997 0.52143
H -2.64715 1.47997 0.52143

***inverted nido*-$[B_{12}H_{12}]^{4-}$**

SCF Energy = -304.771557386
Enthalpy 0K = -304.628354
Enthalpy 298K = -304.617051
Free Energy 298K = -304.661291
Lowest Frequencies = -220.0 116.8 cm^{-1}
B 2.03441 0.00000 0.32250
B 1.20053 -1.41115 0.12300
B 1.20053 1.41115 0.12300
B 1.03148 0.00000 -1.31222
B 0.46828 -0.00000 1.37388
B -0.13168 -1.49478 -1.10161
B -0.13168 1.49479 -1.10161
B -0.51276 1.51859 0.66256
B -0.51277 -1.51859 0.66256
B -1.36745 0.00000 0.99475
B -1.67976 -0.90423 -0.56220
B -1.67976 0.90423 -0.56220
H 3.23353 -0.00000 0.69641
H 1.87294 -2.47652 0.31401
H 1.87294 2.47652 0.31401
H 1.85782 0.00000 -2.26438
H 0.71088 -0.00000 2.59542
H -0.00617 -2.62242 -1.67395
H -0.00617 2.62242 -1.67395
H -0.73852 2.50640 1.39913
H -0.73852 -2.50640 1.39912
H -2.13679 -0.00000 1.98552
H -2.75944 -1.57459 -0.60167
H -2.75944 1.57459 -0.60166

***basket*-$[B_{12}H_{12}]^{4-}$**

SCF Energy = -304.784487828
Enthalpy 0K = -304.639785
Enthalpy 298K = -304.629056
Free Energy 298K = -304.672062
Lowest Frequencies = -97.3 172.1 cm^{-1}
B 1.12356 0.06021 -1.22782
B 0.13797 -1.44277 -0.89370
B -1.45868 -0.98163 -0.25296
B -1.85314 0.73627 -0.32689
B -0.67871 0.02735 -1.56595
B 0.75721 1.78467 -0.43718
B 1.85606 0.73015 0.32664
B 1.45392 -0.98638 0.25339
B -0.14414 -1.44187 0.89457
B -1.12379 0.06555 1.22744
B -0.74827 1.78646 0.43580
B 0.67845 0.02544 1.56599
H 1.10921 2.72020 -1.24840
H -1.00746 -0.09463 -2.77668
H -3.11914 0.81576 -0.39885
H -2.39731 -1.79623 -0.39569
H 0.28677 -2.51363 -1.52006
H 1.88791 -0.03560 -2.21758
H 1.00689 -0.09501 2.77673
H -0.29534 -2.51186 1.52180
H 2.38959 -1.80415 0.39700
H 3.12316 0.79989 0.39958
H -1.88851 -0.02512 2.21737
H -1.09799 2.72310 1.24807
